# Six New Species of *Tomentella* (Thelephorales, Basidiomycota) From Tropical Pine Forests in Central Vietnam

**DOI:** 10.3389/fmicb.2022.864198

**Published:** 2022-04-25

**Authors:** Xu Lu, Ting Cao, Trang Thị Thu Nguyễn, Hai-Sheng Yuan

**Affiliations:** ^1^CAS Key Laboratory of Forest Ecology and Management, Institute of Applied Ecology, Chinese Academy of Sciences, Shenyang, China; ^2^College of Basic Medical Science, Liaoning He’s Medical University, Shenyang, China; ^3^University of the Chinese Academy of Sciences, Beijing, China; ^4^Department of Microbiology, Faculty of Biology and Biotechnology, University of Science, Vietnam National University, Ho Chi Minh City, Vietnam

**Keywords:** ectomycorrhizal fungi, phylogenetic analyses, resupinate thelephoroid fungi, taxonomy, tropical mixed forest

## Abstract

Up to this point, studies on the taxonomy and phylogeny of the basidiomycetous genus *Tomentella* stemmed mainly from the temperate to boreal zones of the Northern hemisphere but were scarce in tropical Asia. In this study, six new species—*T. bidoupensis*, *T. brevisterigmata*, *T. cinereobrunnea*, *T. longiechinula*, *T. stipitobasidia*, and *T. verruculata* from central Vietnam in Southeast Asia—are described and illustrated on the basis of morphological characteristics and molecular phylogenetic analyses of the nuclear ribosomal ITS (internal transcribed spacer: ITS1-5.8S-ITS2) and LSU (large subunit: 28S) markers. Maximum likelihood and Bayesian analyses were used to confirm the phylogenetic positions of these new species and all of them can be well recognized by the macroscopical and anatomical characteristics. The new species and closely related species in the phylogenetic tree, and the new species and morphologically similar species are discussed, whereas the host plant for these new species were speculated on the basis of the phylogenetic analyses and the tree species information of the investigated forests.

## Introduction

The genera *Amaurodon* J. Schröt., *Odontia* Pers., *Pseudotomentella* Svrček, *Tomentellopsis* Hjortstam, and *Tomentella* Pers. ex. Pat. belong to the Thelephoraceae Chevall. of the Thelephorales Corner ex Oberw. of the Basidiomycota R.T. Moore (Larsen, 1974; Kõljalg, 1996). As their common morphological characteristics are resupinate and thin basidiocarps, they were recognized as resupinate thelephoroid fungi by Kõljalg (1996). Species of the group all have their own typical characteristics, such as the light blue basidiocarps of *Amaurodon*, the granulose or hydnoid hymenophoral surface of *Odontia*, the basidiospores with bifurcate warts or spines of *Pseudotomentella*, and the absence of rhizomorphs in *Tomentellopsis*. However, the genus *Tomentella* has diverse and complex morphological features, such as basidiocarps with various colors, basidiospores with diverse shapes and ornamentations, and basidiocarps with smooth to granulose surface (Larsen, 1974; Kõljalg, 1996).

Species in the genus *Tomentella* have been recognized as ectomycorrhizal (ECM) fungi since 1980s (Danielson et al., 1983; Kõljalg et al., 2000; Tedersoo et al., 2014), and the *Tomentella-Thelephora* lineage has been found to be one of the most species-rich, frequent, and abundant groups in a variety of forest ecosystems (Tedersoo et al., 2013; Jakucs et al., 2015; Nouhra et al., 2015). They play an important role in nutrient cycling and ecological functions in forest ecosystems as ectomycorrhiza (Read and Perez-Moreno, 2003; Jakucs et al., 2015; Nouhra et al., 2015). Species of *Tomentella* can form ectomycorrhiza with many host tree families, including Achatocarpaceae, Apocynaceae, Betulaceae, Cistaceae, Dipterocarpaceae, Ericaceae, Fabaceae, Fabaceae subfamily Caesalpinioideae, Fagaceae, Orchidaceae, Pinaceae, Myrtaceae, Nothofagaceae, Nyctaginaceae, Papilionoideae, Polygonaceae, Pyrolaceae, Rhamnaceae, Rosaceae, Salicaceae, Ticodendraceae, and Tiliaceae (Tedersoo et al., 2007, 2008; Smith et al., 2011; Jakucs et al., 2015; Salomón et al., 2017; Alvarez-Manjarrez et al., 2018; Malysheva et al., 2018; Põlme, 2018).

The basidiocarps of *Tomentella* are often found on fallen branches and leaves, decayed coniferous and deciduous wood debris, bark, soil, twigs, stumps, stone, or even charred wood in forests ranging from temperate to tropical zones (Larsen, 1974; Kõljalg, 1996; Kuhar et al., 2016). Approximately 400 names of *Tomentella* are recorded in the databases Index Fungorum and MycoBank, and 1033 species hypothesis codes of this genus were recorded in UNITE database according to threshold value 1.5% (Nilsson et al., 2019). Approximately 200 species accepted in these databases were from the diversity and taxonomic studies based on basidiocarp specimens in countries worldwide, including 91 species from North America (Larsen, 1974); 41 species from British Isles of United Kingdom (Wakefield, 1969); 48 species from temperate Eurasia (Kõljalg, 1996; Lu et al., 2018a,b); 13 species from West Africa (Yorou et al., 2007, 2011, 2012a,b; Yorou, 2008; Yorou and Agerer, 2008); 8 species from the West India (Welden, 1968; Thind and Rattan, 1971); 12 species from Italy (Yorou and Agerer, 2011a,b); 3 species from South America (Kuhar et al., 2016); 1 species from South India (Natarajan and Chandrashekara, 1978); 11 species from Canada, Trinidad, Jamaica, Venezuela, Pakistan, and South Africa (Wakefield, 1966); and 46 and 3 species from Northeast and Northwest China, respectively (Yuan et al., 2020; Lu and Yuan, 2021). In addition, eight tomentelloid species from Australia and New Zealand have also been reported (Cunningham, 1963; Agerer and Bougher, 2001).

At present, few studies on *Tomentella* have been conducted in the regions of Southeast Asia, and only some sequences of Thelephoraceae were reported from ectomycorrhizal samples of *Dipterocarpus alatus* Roxb. and *Hopea odorata* Roxb. seedlings collected from South Vietnam, Malaysia, and Thailand (Ingleby et al., 2000; Sirikantaramas et al., 2003; Kaewgrajang et al., 2014). Sixteen nuclear ribosomal DNA sequences of *Tomentella* spp. from Vietnam are recorded in the UNITE database (Nilsson et al., 2019), but they currently do not match any species names. In this study, morphological characters and phylogenetic analyses of the nuclear ribosomal internal transcribed spacer (ITS) region (ITS1-5.8S-ITS2) and the large subunit (LSU) enable the identification of six undescribed species among the *Tomentella* specimens collected from central Vietnam, and we described them as new species.

## Materials and Methods

### Specimen Collection

Specimens were collected from *Bidoup Nui Ba National Park* in the Central Highlands, Lam Dong Province of central Vietnam (108°17′00′′E–108°42′00′′E, 12°00′00′′N–12°52′00′′N, Altitude: 1450–1505 m), where the mean annual temperature is 18.2°C with an annual rainfall of 1860 mm (Nguyen et al., 2012; Eskov et al., 2019). The native species *Pinus kesiya* is the dominant tree species in the broadleaved forests mixed with Ericaceae, Fagaceae, Lentibulariaceae, Orchidaceae, and Rosaceae of this area (Nguyen et al., 2012; Pócs et al., 2019).

### Morphological Studies

The specimens were deposited in the herbarium of Institute of Applied Ecology, Chinese Academy of Sciences (IFP), and in the private herbarium of Trang Thị Thu Nguyễn. The photos of the basidiocarps were produced with a camera (Canon 5D Mark III: Tokyo, Japan). Macromorphological characteristics including the color, texture, and thickness of basidiocarps, hymenophoral surface, and sterile margin were examined under a *stereomicroscope* (*Nikon* SMZ 1000: Tokyo, Japan) at 4× magnification. Special color terms followed Kornerup and Wanscher (1981) for the macromorphological description. The microscopic procedure follows Lu et al. (2018b) with some minor amendments. Cyanophilous or acyanophilous reactions were assessed using Cotton Blue. Amyloid and dextrinoid reactions were tested using Melzer’s reagent. The following abbreviations were used in the text: KOH = 2.5% potassium hydroxide, inamyloid = neither amyloid nor dextrinoid, *L* = mean spore length (arithmetic average of all spores in lateral face), *W* = mean spore width (arithmetic average of all spores in lateral face), *Q* = variation in the ratios of *L*/*W* between specimens studied, a *n* = number of spores measured from a given number of specimens. For spore measurements, and the ornamentation was excluded. Micromorphological descriptions were studied at magnifications up to 1000× with a light microscope (Nikon Eclipse E600: Tokyo, Japan) with phase contrast illumination, and dimensions were estimated subjectively with an accuracy of 0.2 μm. Drawings were made with the aid of a drawing tube. The surface morphology for the basidiospores was observed with a QUANTA 250 scanning electron microscope (ESEM, QUANTA 250, FEI, Netherlands) at an accelerating voltage of 25 kV. The working distance was 12.2 mm. A thin layer of gold was coated on the samples to avoid charging.

### Molecular Procedures and Phylogenetic Analyses

Total genomic DNA was extracted from the dried specimens with a Thermo Scientific Phire Plant Direct PCR Kit (Thermo Fisher Scientific, Waltham, MA, United States). PCR reactions were performed in 30 μl of reaction mixtures containing 15 μl of 2 × Phire^®^ Plant PCR buffer, 0.6 μl of Phire^®^ Hot Start II DNA Polymerase, 1.5 μl of each PCR primer (10 μM), 10.5 μl of doubly deionized H_2_O (ddH_2_O), and 0.9 ml of template DNA. The ITS region was amplified with the primers SSU1318-Tom (5′-CGATAACGAACGAGACCTTAT-3′) and LSU-Tom4 (5′-GCCCTGTTCCAAGAGACTTA-3′) (Taylor and McCormick, 2008). The LSU gene was amplified with the primers LROR (5′-ACCCGCTGAACTTAAGC-3′) and LR7 (5′-TACTACCACCAAGATCT-3′) (Vilgalys and Hester, 1990; Moncalvo et al., 1993).

The PCR thermal cycling program conditions were as follows: initial denaturation at 95°C for 5 min, followed by 39 cycles at 95°C for 30 s, ×°C (the annealing temperatures for SSU1318-Tom/LSU-Tom4 and LROR/LR7 were 62°C and 47.2°C, respectively) for 30 s (Vilgalys and Hester, 1990; Taylor and McCormick, 2008), at 72°C for 20 s, and a final extension at 72°C for 1 min. PCR reaction products were confirmed by 1% agarose electrophoresis gels stained with ethidium bromide (Li et al., 1994) and sequenced at the Beijing Genomics Institute (BGI) with the same primers. The sequences were assembled with the software DNAMAN 8 (Lynnon Biosoft, Pointe-Claire, QB, Canada) and modified manually. The basic integrity and quality control of all newly generated sequences were verified and exercised (Nilsson et al., 2012). The newly generated ITS and LSU nuclear rDNA sequences were deposited in GenBank (Supplementary Table 1). The 205 related sequences from GenBank (Benson et al., 2018) and UNITE were used for the phylogenetic analyses (Supplementary Table 1). ITS and LSU sequences were aligned with Muscle in MEGA 5.02 separately (Tamura et al., 2011), using default settings and further manually optimized to allow maximum alignment and minimize gaps, and deposited in TreeBASE (study no. 24255). FASTA alignment formats were converted into NEXUS and PHYLIP files in ClustalX and EasyCodeMLv1.2 (Gao et al., 2019), respectively.

Our phylogenetic analyses of the combined ITS–LSU dataset used maximum likelihood (ML) and Bayesian approaches. ML analysis was performed in RAxMLGUI1.5b1 with the GTRGAMMA model and 100 rapid bootstrap replicates (Silvestro and Michalak, 2012). Bayesian analysis was conducted in MrBayes 3.2.6 (Ronquist and Huelsenbeck, 2003) implementing the Markov Chain Monte Carlo technique. For the Bayesian analyses, the combined dataset was divided into four partitions: ITS1 as subset 1, 5.8S as subset 2, ITS2 as subset 3, and LSU as subset 4. The HKY + G model for subsets 1 and 3 and GTR + I + G model for subsets 2 and 4 were proposed by jModelTest 2.1.10 (Darriba et al., 2012) on the basis of the corrected Akaike information criterion. Four simultaneous Markov chains were run starting from random trees and keeping one tree every 100th generation until the average standard deviation of split frequencies was below 0.01. The burn-in was set to discard 25% of trees when calculating the posterior probabilities. Bayesian posterior probabilities were obtained from the 50% majority rule consensus of the trees kept.

## Results

### Phylogenetic Analyses

**FIGURE 1:**
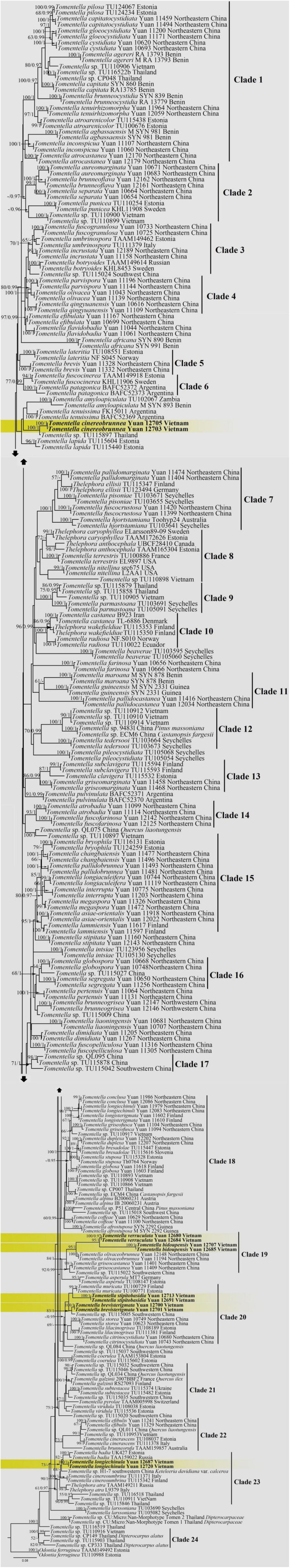
Maximum likelihood tree illustrating the phylogeny of *Tomentella bidoupensis*, *T. brevisterigmata*, *T. cinereobrunnea*, *T. longiechinula*, *T. stipitobasidia*, *T. verruculata*, and related taxa, based on ITS + LSU nuclear rDNA sequences dataset. Branches are labeled with maximum likelihood bootstrap equal to or higher than 50% and Bayesian posterior probabilities equal to or higher than 0.95. The terminals indicate the regions where they were distributed or the hosts of the *Tomentella* species. New species in bold (black).

The combined dataset of the 276 sequences had an aligned length of 1683 sites with 643-bp ITS and 1040-bp LSU, which included 955 constant characters, 109 parsimony-uninformative variable characters, and 619 parsimony informative positions. The multiple sequence alignment included the following: 12 sequences of the 6 new species; 10 sequences of 5 *Thelephora* species (Kõljalg et al., 2000; Tedersoo et al., 2014; Larsson et al., 2019); 199 sequences of 101 other *Tomentella* species (Larsson et al., 2004; Tedersoo et al., 2006; Smith et al., 2007; Yorou et al., 2007, 2011; Morris et al., 2008; Yorou and Agerer, 2008; Suvi et al., 2010; Richard et al., 2011; Ge et al., 2012; Peintner and Dämmrich, 2012; Huang et al., 2014; Disyatat et al., 2016); 53 sequences of *Tomentella* spp. from China, Vietnam, and Thailand (Wang et al., 2011, 2012; Ge et al., 2012; Huang et al., 2014; Kaewgrajang et al., 2014; Tedersoo et al., 2014; Disyatat et al., 2016); and 2 outgroup sequences of *Odontia ferruginea* from Estonia (Tedersoo et al., 2014; Yuan et al., 2018). The Bayesian analyses ran for 20 million generations and resulted in an average standard deviation of split frequencies of 0.007989. The ML and Bayesian analyses produced a similar topology, and the ML tree is shown in Figure 1. In the phylogenetic tree, the ingroup species were divided into 24 main clades, of which 17 clades (clade 1, clade 3, clade 4, clade 5, clade 7, clade 8, clade 10, clade 11, clade 13, clade 14, clade 15, clade 18, clade 19, clade 20, clade 21, clade 22, and clade 23) are consistent with the previous ITS phylogenetic analyses (Yuan et al., 2020). Nine of the 17 clades are more strongly supported (100% ML/1.00 BPP for clade 7, 86% ML/0.99 BPP for clade 13, 93% ML/1.00 BPP for clade 14, 60% ML/0.99 BPP for clade 18, 84% ML/1.00 BPP for clade 19 and 0.95 BPP for clade 20, 75% ML/1.00 BPP for clade 21, 73% ML/1.00 BPP for clade 22, and 61% ML/0.97 BPP for clade 23, respectively), and the others lacked significant support. In addition, 7 other clades of the 24 main clades enjoyed moderate or strong support (0.97 BPP for clade 2, 77% ML/0.99 BPP for clade 6, 100% ML/1.00 BPP for clade 9, 70% ML/0.99 BPP for clade 12, 68% ML/1.00 BPP for clade 16, 71% ML/1.00 BPP for clade 17, and 52% ML for clade 24, respectively). However, support at deeper nodes of the phylogenetic tree was generally weak.

The 12 sampled specimens of these 6 new species formed 6 single subclades with full support (100% ML/0.97 BPP for *T. verruculata* and 100% ML/1.00 BPP for other species) in the tree, respectively. Four new species have distributed in clade 19 and clade 20, which did not contain any other sequences from Vietnam recorded in UNITE database. One new species (*T. longiechinula*) distributed in clade 23, which also include one sequences (TU110911) from Vietnam and two sequences (TU116518 and TU115846) from Thailand. In addition, the other new species (*T. cinereobrunnea*) also clustered with one sequence (TU115897) from Thailand with strong support (100% ML/1.00 BPP). The phylogenetic tree also shows that every *Tomentella* species can associate with different host tree species in different families: *Pinus massoniana* in Pinaceae and *Castanopsis fargesii* in Fagaceae (specimens ECM6 and 94831).

### Taxonomy

***Tomentella bidoupensis*** X. Lu and H.S. Yuan, sp. nov.

**FIGURE 2:**
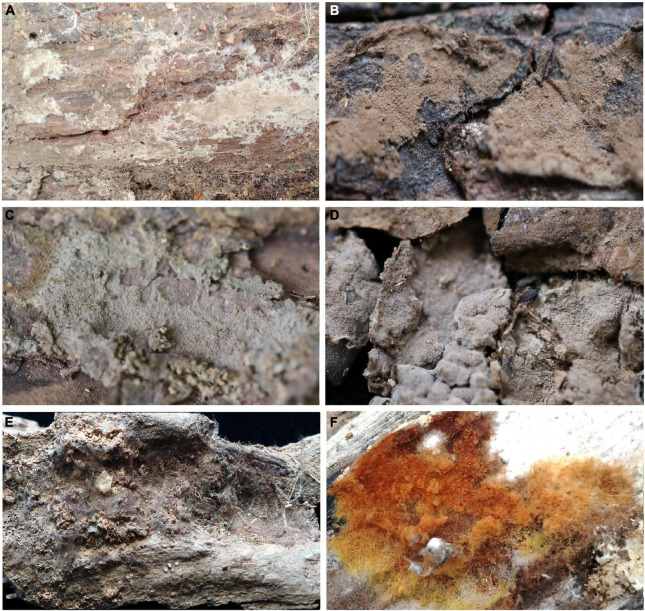
Basidiocarps of *Tomentella* species. **(A)** *T. bidoupensis* (holotype Yuan 12707); **(B)** *T. brevisterigmata* (holotype Yuan 12700); **(C)** *T. cinereobrunnea* (holotype Yuan 12703); **(D)** *T. longiechinula* (holotype Yuan 12687); **(E)** *T. stipitobasidia* (holotype Yuan 12713); **(F)** *T. verruculata* (holotype Yuan 12684).

**FIGURE 3:**
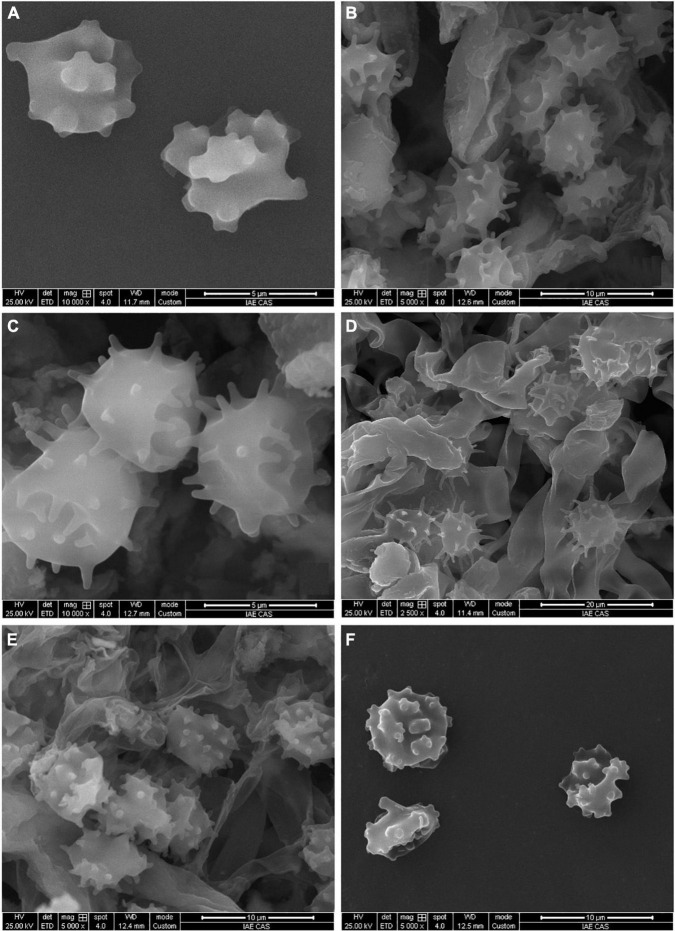
SEM of basidiospores of *Tomentella* species. **(A)** *T. bidoupensis* (holotype Yuan 12707); **(B)** *T. brevisterigmata* (holotype Yuan 12700); **(C)** *T. cinereobrunnea* (holotype Yuan 12703); **(D)** *T. longiechinula* (holotype Yuan 12687); **(E)** *T. stipitobasidia* (holotype Yuan 12713); **(F)** *T. verruculata* (holotype Yuan 12684).

**FIGURE 4:**
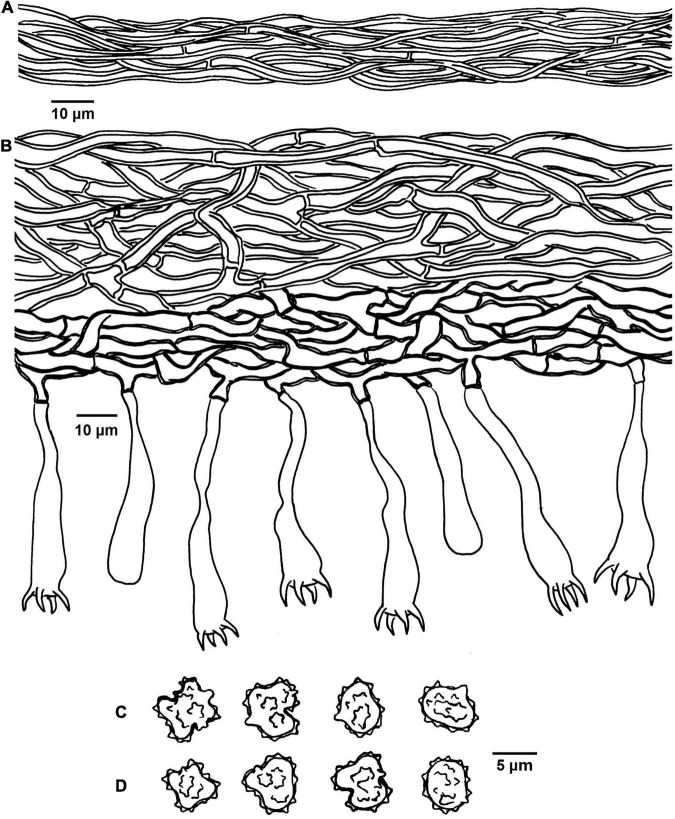
Microscopic structures of *Tomentella bidoupensis* (drawn from holotype Yuan 12707). **(A)** Hyphae from a rhizomorph. **(B)** A section through basidiocarp. **(C)** Basidiospores in lateral view. **(D)** Basidiospores in frontal view.

MycoBank no. MB830545 (Figures 2A, 3A, 4).

Diagnosis: hymenophoral surface: orange-gray to grayish brown; sterile margin: whitish; hyphae in rhizomorphs: simple-septate; generative hyphae: simple-septate; basidiospores: verruculose and warts up to 0.5 μm long.

Type: VIETNAM, Lam dong Province: Lac Duong District, Bidoup Nui Ba National Park, 12°11′08′′N, 108°40′41′′E, 1495 m, on rotten wood debris of *P. kesiya*, October 16, 2017, Yuan 12707 (holotype: IFP 019342, GenBank ITS: MK775477; LSU: MN684329; UNITE SH: SH1502340.08FU).

Etymology. *Bidoupensis* (Lat.), referring to the distribution of the type specimen.

Basidiocarps: annual, resupinate, separable from the substrate, mucedinoid, without odor or taste when fresh, 0.3–0.6 mm thick, continuous. Hymenophoral surface: smooth, or range-gray to grayish brown (5B2–5E3) and turning darker than subiculum. Sterile margin: determinate, byssoid, whitish, paler than hymenophore.

Rhizomorphs: present in subiculum and margins, 8–15 μm diameter; rhizomorphic surface: more or less smooth; hyphae in rhizomorph: monomitic, undifferentiated, of type B (according to Agerer, 1987-2008), compactly arranged and uniform; single hyphae: simple-septate, thick-walled, unbranched, 1–2 μm diameter, grayish brown in KOH, cyanophilous, inamyloid.

Subicular hyphae: monomitic; generative hyphae: simple-septate, thick-walled, 3–5 μm diameter, without encrustation and grayish brown in KOH and distilled water, cyanophilous, inamyloid. Subhymenial hyphae: simple-septate, slightly thick-walled, 2–6 μm diameter, without encrustation; hyphal cells: short, grayish brown in KOH and in distilled water, cyanophilous, inamyloid.

Cystidia: absent.

Basidia: 20–55 μm long and 5–9 μm diameter at apex, 1.5–2 μm at base, with simple septa at base, utriform, not stalked, sinuous, rarely with transverse septa, grayish brown in KOH and distilled water, 4-sterigmata; sterigmata: 5–8 μm long and 1–1.5 μm diameter at base.

Basidiospores: slightly thick-walled, (5.5–)6–7(–7.5) × (5–)5.5–6.5(–7) μm, *L* = 6.78 μm, *W* = 5.98 μm, *Q* = 1.12–1.16 (*n* = 60/2), subglobose to bi-, tri-, or quadra-lobed in frontal and later face, verruculose, grayish brown in KOH and distilled water, cyanophilous, inamyloid; warts usually grouped in 2 or more, up to 0.5 μm long.

Additional specimens (paratype) examined: VIETNAM, Lam dong Province: Lac Duong District, *Bidoup Nui Ba National Park*, 12°11′35′′N, 108°40′32′′E, 1455 m, on rotten wood debris of *P. kesiya*, 16 X. 2017, Yuan 12685 (IFP 019335, GenBank ITS: MK775476; LSU: MN684330).

Notes: *T. bidoupensis* and *T. verruculata* formed a close relationship with a significant support (95% in ML and 1.00 BPP) in the phylogenetic tree (Figure 1), and the resupinate, continuous basidiocarps separable from the substrate, byssoid sterile margin, simple-septate hyphae, rhizomorphs with the same type, presence of the monomitic rhizomorphs and the basidiospores of approximately the same shape and size are their common characteristics. However, *T. verruculata* is differentiated from *T. bidoupensis* by arachnoid basidiocarps and comparatively narrower hyphae of rhizomorphs (3–4 μm in diameter). The clade 19 of the tree (Figure 1) also contains *T. olivaceobrunnea* and *T. griseocastanea*, and the clamped hyphae and the absence of rhizomorphs of them can be obviously distinguished from the *T. bidoupensis* (Yuan et al., 2020).

***Tomentella brevisterigmata*** X. Lu and H.S. Yuan, sp. nov.

**FIGURE 5:**
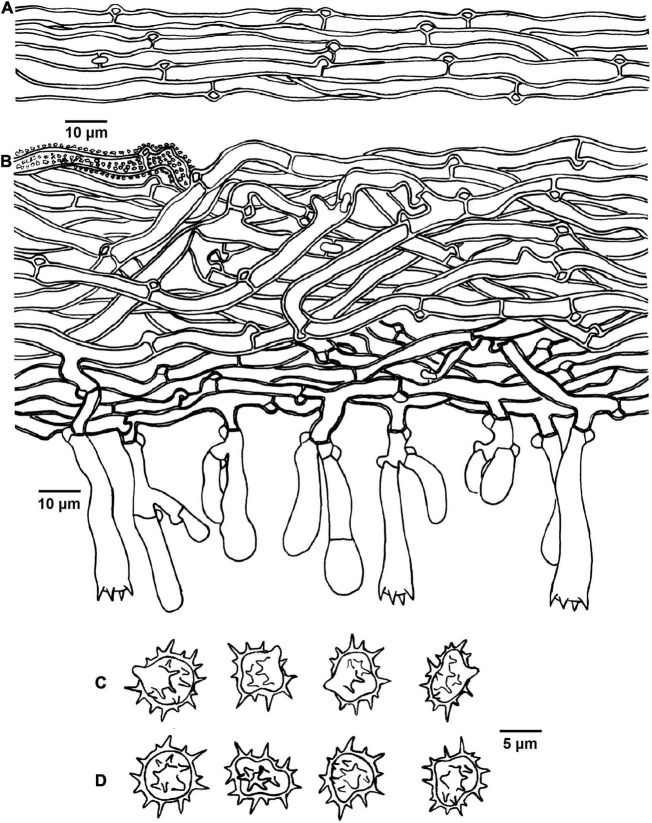
Microscopic structures of *Tomentella brevisterigmata* (drawn from holotype Yuan 12700). **(A)** Hyphae from a rhizomorph. **(B)** A section through basidiocarp. **(C)** Basidiospores in lateral view. **(D)** Basidiospores in frontal view.

MycoBank no. MB830546 (Figures 2B, 3B, 5).

Diagnosis: hymenophoral surface: grayish brown to dark brown; sterile margin: grayish brown; hyphae in rhizomorphs: clamped; basidia: short sterigmata; basidiospores: echinuli up to 2 μm long.

Type: VIETNAM, Lam dong Province: Lac Duong District, Bidoup Nui Ba National Park, 12°11′05′′N, 108°40′40′′E, 1476 m, on fallen wood debris of *P. kesiya*, October 16, 2017, Yuan 12700 (holotype: IFP 019338, GenBank ITS: MK775472; LSU: MK850202; UNITE SH: SH1502687.08FU).

Etymology. *Brevisterigmata* (Lat.), referring to the basidia with short sterigmata.

Basidiocarps: annual, resupinate, separable from the substrate, arachnoid, without odor or taste when fresh, 0.5–0.8 mm thick, continuous. Hymenophoral surface: smooth, grayish brown to dark brown (6D3–6F5) and concolorous with the subiculum. Sterile margin: often indeterminate, byssoid, concolorous with hymenophore.

Rhizomorphs: present in subiculum and margins: 10–20 μm diameter; rhizomorphic surface: more or less smooth; hyphae in rhizomorph: monomitic, undifferentiated, of type B (according to Agerer, 1987-2008), compactly arranged and uniform; single hyphae: clamped, thick-walled, unbranched, 4–6 μm diameter, brown in KOH, cyanophilous, inamyloid.

Subicular hyphae: monomitic; generative hyphae: clamped and rarely simple-septate, thick-walled, 3–5 μm diameter, with encrustation, brown in KOH and distilled water, cyanophilous, inamyloid. Subhymenial hyphae: clamped and rarely simple-septate, slightly thick-walled, 2.5–5 μm diameter, without encrustation; hyphal cells: more or less uniform, grayish brown in KOH and in distilled water, cyanophilous, inamyloid.

Cystidia: absent.

Basidia: 20–65 μm long and 4–9 μm diameter at apex, 2.5–4 μm at base, with a clamp connection at base, utriform, not stalked, sinuous, rarely with transverse septa, grayish brown in KOH and distilled water, 4-sterigmata; sterigmata: 2–3 μm long and 1.5–2 μm diameter at base.

Basidiospores: thick-walled, (6–)7–8(–8.5) × (5–)5.5–7(–7.5) μm, *L* = 7.24 μm, *W* = 5.92 μm, *Q* = 1.15–1.28 (*n* = 60/2), subglobose to bi-, tri-, or quadra-lobed in frontal and later face, echinulate to aculeate, grayish brown in KOH and distilled water, cyanophilous, inamyloid; echinuli usually grouped in 2 or more, up to 2 μm long.

Additional specimens (paratype) examined: VIETNAM, Lam dong Province: Lac Duong District, *Bidoup Nui Ba National Park*, 12°11′09′′N, 108°40′43′′E, 1489 m, on fallen wood debris of *P. kesiya*, October 16, 2017, Yuan 12701 (IFP 019339, GenBank ITS: MK775473; LSU: MK850203).

Notes: In the phylogenetic tree (Figure 1), *T. brevisterigmata*, *T. stipitobasidia*, *T. muricata*, and one sequences (TU115005, ECM) from Southwestern China clustered together without support. They exhibit some similar characteristics: arachnoid basidiocarps, more or less similar color of the hymenophoral surface (brown, grayish brown, or dark brown), monomitic rhizomorphs with clamped hyphae, clamped generative hyphae and utriform basidia (Kõljalg, 1996). However, *T. muricata* presents clavate cystidia and larger basidiospores (8.5–9.5 μm) (Kõljalg, 1996). In addition, *T. stipitobasidia* differs from *T. brevisterigmata* by the slightly thick-walled basidiospores with shorter echinuli (1 μm long).

***Tomentella cinereobrunnea*** X. Lu and H.S. Yuan, sp. nov.

**FIGURE 6:**
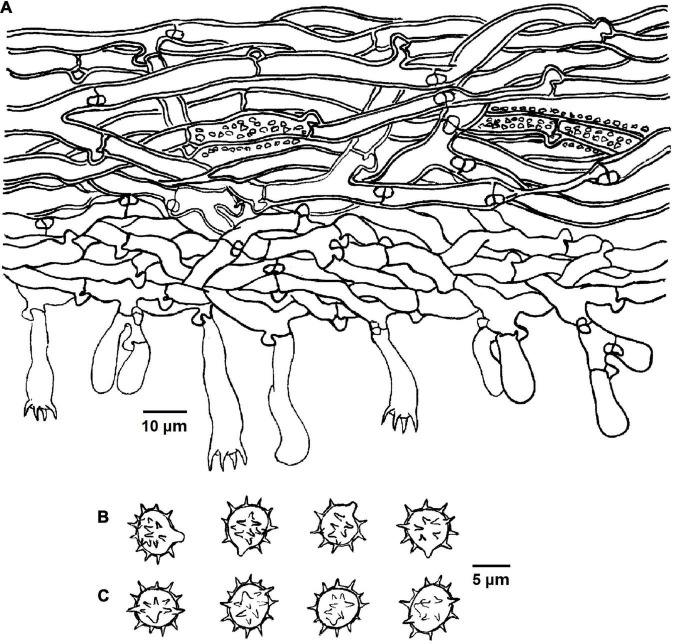
Microscopic structures of *Tomentella cinereobrunnea* (drawn from Yuan 12703). **(A)** A section through basidiocarp. **(B)** Basidiospores in lateral view. **(C)** Basidiospores in frontal view.

MycoBank no. MB830547 (Figures 2C, 3C, 6).

Diagnosis: hymenophoral surface: grayish brown to brown; sterile margin: grayish brown; generative hyphae: often with encrustation; rhizomorphs: absent; basidiospores: echinuli or aculei up to 1.5 μm long.

Type: VIETNAM, Lam dong Province: Lac Duong District, Bidoup Nui Ba National Park, 12°11′05′′N, 108°40′40′′E, 1478 m, on fallen wood debris of *P. kesiya*, October 16, 2017, Yuan 12703 (holotype: IFP 019340, GenBank ITS: MK775478; LSU: MK850198; UNITE SH: SH1502946.08FU).

Etymology. *Cinereobrunnea* (Lat.), referring to the grayish brown to brown hymenophoral surface.

Basidiocarps: annual, resupinate, separable from the substrate, arachnoid, without odor or taste when fresh, 0.3–0.8 mm thick, continuous. Hymenophoral surface: smooth, grayish brown to brown (5D3–6E6) and concolorous with the subiculum. Sterile margin: often indeterminate, byssoid, concolorous with hymenophore.

Rhizomorphs: absent.

Subicular hyphae: monomitic; generative hyphae: clamped and rarely simple-septate, thick-walled, 4–7 μm diameter, often with encrustation, pale brown in KOH and distilled water, cyanophilous, inamyloid. Subhymenial hyphae: clamped and rarely simple-septate, thin-walled, 3–5 μm diameter, without encrustation; hyphal cells: more or less uniform, pale brown in KOH and in distilled water, acyanophilous, inamyloid.

Cystidia: absent.

Basidia: 15–35 μm long and 4–6 μm diameter at apex, 3–4 μm at base, with a clamp connection at base, utriform, not stalked, sinuous, rarely with transverse septa, pale brown in KOH and distilled water, 4-sterigmata; sterigmata: 4–6.5 μm long and 1–1.5 μm diameter at base.

Basidiospores: slightly thick-walled, (5.5–)6–7(–8) × (5–)5.5–6.5(–7.5) μm, *L* = 6.38 μm, *W* = 5.98 μm, *Q* = 1.05–1.08 (*n* = 60/2), subglobose to globose in frontal and later face, echinulate to aculeate, pale brown in KOH and distilled water, cyanophilous, inamyloid; echinuli or aculei usually isolated, up to 1.5 μm long.

Additional specimens (paratype) examined: VIETNAM, Lac Duong District, Bidoup Nui Ba National Park, 12°11′12′′N, 108°40′41′′E, 1485 m, on fallen wood debris of *P. kesiya*, October 16, 2017, Yuan 12705 (IFP 019341, GenBank ITS: MK775479; LSU: MK850199).

Notes: *T. cinereobrunnea*, *T. amyloapiculata*, *T. tenuissima*, and *T.* sp. (TU115879, ECM) from Thailand belonged to a subclade without support in the phylogenetic tree (Figure 1), and the absence of rhizomorphs and cystidia is their common characteristics. In addition, *T. tenuissima* also possesses encrusted and clamped hyphae that the same as those of *T. cinereobrunnea*, but *T. tenuissima* differs by the blackish hymenophoral surface when dry and larger basidiospores (7.7–9.2 μm) (Kuhar et al., 2016). *T. amyloapiculata* presents three obvious different characteristics comparing with *T. cinereobrunnea*: the crustose basidiocarps, simple-septate hyphae, and the larger basidiospores (8.5–11.5 × 7.5–8.5 μm) (Yorou et al., 2012a).

***Tomentella longiechinula*** X. Lu and H.S. Yuan, sp. nov.

**FIGURE 7:**
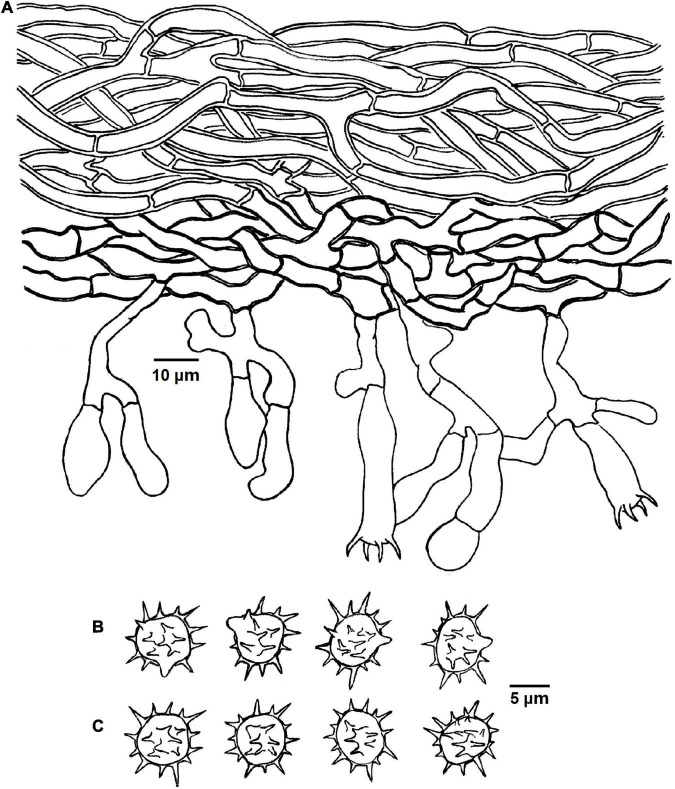
Microscopic structures of *Tomentella longiechinula* (drawn from Yuan 12687). **(A)** A section through basidiocarp. **(B)** Basidiospores in lateral view. **(C)** Basidiospores in frontal view.

MycoBank no. MB830548 (Figures 2D, 3D, 7).

Diagnosis: hymenophoral surface: grayish brown to brown; sterile margin: grayish brown; rhizomorphs: absent; basidiospores: echinuli or aculei up to 4 μm long.

Type: VIETNAM, Lam dong Province: Lac Duong District, Bidoup Nui Ba National Park, on carbonized wood debris of *P. kesiya*, 12°11′35′′N, 108°40′32′′E, 1455 m, October 16, 2017, Yuan 12687 (holotype IFP 019336, GenBank ITS: MK775474; LSU: MK850201; UNITE SH: SH2714357.08FU).

Etymology. *Longiechinula* (Lat.), referring to the long echinuli of basidiospores.

Basidiocarps: annual, resupinate, separable from the substrate, arachnoid, without odor or taste when fresh, 0.8–1.2 mm thick, continuous. Hymenophoral surface: smooth, grayish brown to dark brown (6D3–6F6) and concolorous with the subiculum. Sterile margin: often indeterminate, byssoid, concolorous with hymenophore.

Rhizomorphs: absent.

Subicular hyphae: monomitic; generative hyphae simple-septate, thick-walled, 4–6 μm diameter, without encrustation, pale brown in KOH and distilled water, cyanophilous, inamyloid. Subhymenial hyphae: simple-septate, slightly thick-walled, 4–7 μm diameter, without encrustation; hyphal cells: short, pale brown in KOH and in distilled water, cyanophilous, inamyloid.

Cystidia: absent.

Basidia: 20–55 μm long and 4–12 μm diameter at apex, 3–4 μm at base, with simple septa at base, utriform, not stalked, sinuous, rarely with transverse septa, pale brown in KOH and distilled water, 4-sterigmata; sterigmata: 4–6 μm long and 1–1.5 μm diameter at base.

Basidiospores: slightly thick-walled, (7.5–)8–9(–9.5) × (6.5–)7–8(–8.5) mm, *L* = 8.52 mm, *W* = 7.56 mm, *Q* = 1.05–1.09 (*n* = 60/2), subglobose to globose in frontal and later face, echinulate to aculeate, pale brown in KOH and distilled water, cyanophilous, inamyloid; echinuli or aculei usually grouped in 2 or more, up to 4 μm long.

Additional specimens (paratype) examined: VIETNAM, Lam dong Province: Lac Duong District, Bidoup Nui Ba National Park, 12°11′53′′N, 108°40′55′′E, 1472 m, on carbonized wood debris of *P. kesiya*, October 16, 2017, Yuan 12720 (IFP 019344, GenBank ITS: MK775475; LSU: MK850200).

Notes: *T. longiechinula* and *T. badia* formed a moderately supported relationship (82% in ML) in the phylogenetic tree (Figure 1), and they also clustered with *T. cinereoumbrina* from Finland and *T.* sp. (H1_7) from China with a moderate support (68% in ML). Their common features are the grayish brown to dark brown hymenophoral surface, simple-septate hyphae, utriform basidia, and the absence of rhizomorphs and cystidia. However, *T. badia* is differentiated from *T. longiechinula* by its mucedinoid basidiocarps and larger basidiospores (8–11 μm) with shorter echinuli (1–1.5 μm) (Kõljalg, 1996). *T. cinereoumbrina* differs from *T. badia* and *T. longiechinula* by the crustose basidiocarps (Kõljalg, 1996).

***Tomentella stipitobasidia*** X. Lu and H.S. Yuan, sp. nov.

**FIGURE 8:**
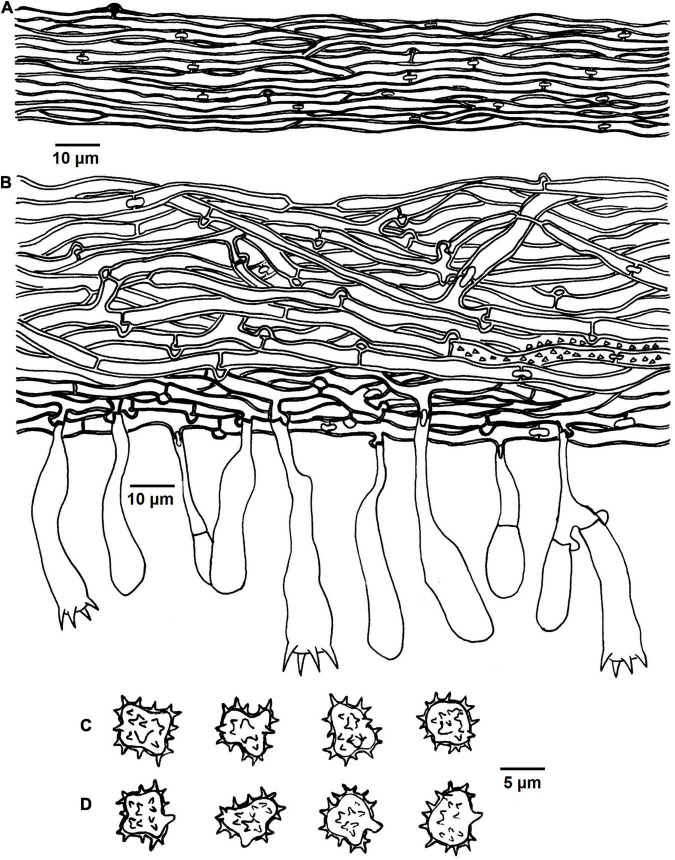
Microscopic structures of *Tomentella stipitobasidia* (drawn from Yuan 12713). **(A)** Hyphae from a rhizomorph. **(B)** A section through basidiocarp. **(C)** Basidiospores in lateral view. **(D)** Basidiospores in frontal view.

MycoBank no. MB830549 (Figures 2E, 3E, 8).

Diagnosis: hymenophoral surface: brown to dark brown; sterile margin: brown; generative hyphae: rarely with encrustation; basidia: stipe or little stalk with a clamp connection at base; basidiospores: echinuli up to 1 μm long.

Type: VIETNAM, Lam dong Province: Lac Duong District, Bidoup Nui Ba National Park, 12°11′52′′N, 108°40′55′′E, 1455 m, on wood debris of *P. kesiya*, October 16, 2017, Yuan 12713 (holotype IFP 019343, GenBank ITS: MK775470; LSU: MK850204; UNITE SH: SH1502937.08FU).

Etymology. *Stipitobasidia* (Lat.), referring to the basidia with a stipe or little stalk.

Basidiocarps: annual, resupinate, separable from the substrate, arachnoid, without odor or taste when fresh, 0.5–1 μm thick, continuous. Hymenophoral surface: smooth, brown to dark brown (7E5–7F6) and concolorous with the subiculum. Sterile margin: often determinate, byssoid, concolorous with hymenophore.

Rhizomorphs: present in subiculum and margins, 60–75 μm diameter; rhizomorphic surface: more or less smooth; hyphae in rhizomorph: monomitic, undifferentiated, of type B (according to Agerer, 1987-2008), compactly arranged and uniform; single hyphae: clamped, slightly thick-walled, unbranched, 2–3 μm diameter, brown in KOH, cyanophilous, inamyloid.

Subicular hyphae: monomitic; generative hyphae: clamped and rarely simple-septate, thick-walled, 2–6 μm diameter, occasionally collapsed, rarely with encrustation, brown in KOH and distilled water, cyanophilous, inamyloid. Subhymenial hyphae: clamped and rarely simple-septate, slightly thick-walled, 2.5–5 μm diameter, without encrustation; hyphal cells: more or less uniform, brown in KOH and in distilled water, cyanophilous, inamyloid.

Cystidia: absent.

Basidia: 30–60 μm long and 6–12 μm diameter at apex, 2–3.5 μm at base, with a clamp connection at base, utriform, stipe or little stalk, sinuous, rarely with transverse septa, brown in KOH and distilled water, 4-sterigmata; sterigmata: 4–6 μm long and 1.5–2 μm diameter at base.

Basidiospores: slightly thick-walled, (5.5–)6.5–8(–8.5) × (4–)4.5–7(–7.5) μm, *L* = 6.9 μm, *W* = 5.76 μm, *Q* = 1.09–1.36 (*n* = 60/2), subglobose to bi-, tri-, or quadra-lobed in frontal and later face, echinulate, brown in KOH and distilled water, cyanophilous, inamyloid; echinuli usually grouped in 2 or more, up to 1 μm long.

Additional specimens (paratype) examined: VIETNAM, Lam dong Province: Lac Duong District, Bidoup Nui Ba National Park, 12°11′45′′N, 108°40′47′′E, 1455 m, on wood debris of *P. kesiya*, October 16, 2017, Yuan 12691 (IFP 019337, GenBank ITS: MK775471; LSU: MK850205).

Notes: In the phylogenetic tree, *T. stipitobasidia* clustered with *T. storea* and *T. citrinocystidiata* from Northeastern China and *T. lilacinogrisea* from Europe, and these species share the similar color of the hymenophoral surface (Wakefield, 1966; Yuan et al., 2020). In addition, the encrusted subicular hyphae and presence of rhizomorphs for *T. lilacinogrisea* are also similar to *T. stipitobasidia*, and the adherent basidiocarps are the opposite of *T. stipitobasidia* (Wakefield, 1966). However, *T. storea* can be distinguish from *T. stipitobasidia* by the mat-like basidiocarps and absence of the rhizomorphs, and the pelliculose basidiocarps and capitate cystidia of *T. citrinocystidiata* can set it apart from *T. stipitobasidia* (Yuan et al., 2020).

***Tomentella verruculata*** X. Lu and H.S. Yuan, sp. nov.

**FIGURE 9:**
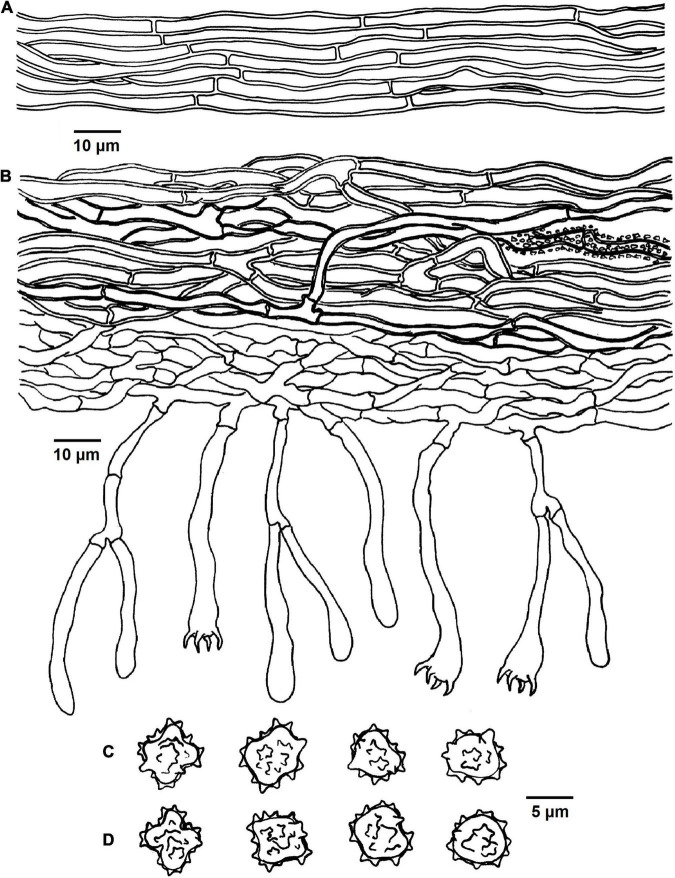
Microscopic structures of *Tomentella verruculata* (drawn from Yuan 12684). **(A)** Hyphae from a rhizomorph. **(B)** A section through basidiocarp. **(C)** Basidiospores in lateral face. **(D)** Basidiospores in frontal view.

MycoBank no. MB830550 (Figures 2F, 3F, 9).

Diagnosis: hymenophoral surface: light brown to dark brown; sterile margin: light brown; hyphae in rhizomorphs: simple-septate; generative hyphae: simple-septate; basidiospores: verruculose and warts up to 1 μm long.

Type: VIETNAM, Lam dong Province (Lat.), Lac Duong District, Bidoup Nui Ba National Park, 12°11′35′′N, 108°40′32′′E, 1465 m, on wood debris of *P. kesiya*, October 16, 2017, Yuan 12684 (holotype IFP 019334, GenBank ITS: MK775469; LSU: MN684332; UNITE SH: SH1528455.08FU).

Etymology. *Verruculata* (Lat.), referring to the verruculose basidiospores.

Basidiocarps: annual, resupinate, separable from the substrate, arachnoid, without odor or taste when fresh, 0.5–1 mm thick, continuous. Hymenophoral surface: smooth, light brown to dark brown (6D6–6F8) and concolorous with the subiculum. Sterile margin: often determinate, byssoid, concolorous with hymenophore.

Rhizomorphs: present in subiculum and margins, 5–18 μm diameter; rhizomorphic surface: more or less smooth; hyphae in rhizomorph: monomitic, undifferentiated, of type B (according to Agerer, 1987-2008), compactly arranged and uniform; single hyphae: simple-septate, thick-walled, unbranched, 3–4 μm in diameter, pale brown in KOH, cyanophilous, inamyloid.

Subicular hyphae: monomitic; generative hyphae: simple-septate, slightly thick- to thick-walled, 3–5 μm diameter, without encrustation, pale to dark brown in KOH and distilled water, cyanophilous, inamyloid. Subhymenial hyphae: simple-septate, thin-walled, 2.5–3.5 μm diameter, without encrustation; hyphal cells: more or less uniform, pale to dark brown in 2.5% KOH and in distilled water, acyanophilous, inamyloid.

Cystidia: absent.

Basidia: 35–55 μm long and 4–7.5 μm diameter at apex, 2–3.5 μm at base, with simple septa at base, clavate, stalked, sinuous, without transverse septa, pale brown in KOH and distilled water, 4-sterigmata; sterigmata: 4–6 μm long and 1–1.5 μm diameter at base.

Basidiospores: slightly thick-walled, (6–)6.5–7.5(–8) × (5–)5.5–6(–6.5) μm, *L* = 6.78 μm, *W* = 5.72 μm, *Q* = 1.19–1.27 (*n* = 60/2), subglobose to bi-, tri-, or quadra-lobed in frontal and later face, verruculose, pale brown in KOH and distilled water, cyanophilous, inamyloid; warts usually grouped in 2 or more, up to 1 μm long.

Additional specimens (paratype) examined: VIETNAM, Lam dong Province: Lac Duong District, Bidoup Nui Ba National Park, Giang Ly, 12°11′32′′N, 108°40′28′′E, 1465 m, on fallen branch of *P. kesiya*, October 15, 2017, Yuan 12680 (IFP 019333, GenBank ITS: MK775468; LSU: MN684331).

Notes: *T. verruculata*, *T. bidoupensis*, *T. olivaceobrunnea*, *T. griseocastanea*, and *T. asperula* clustered together in the clade 19 of the phylogenetic tree. Whereas, *T. verruculata* and *T. bidoupensis* possess significant morphological differences in comparison with *T. olivaceobrunnea* and *T. griseocastanea*. The latter two species can be distinctly differentiated from *T. verruculata* by the basidiocarps adherent to the substrate, granulose sterile margin, clamped hyphae with rare simple septa and the absence of the rhizomorphs. *T. asperula* is similar to *T. verruculata* by the separable basidiocarps and presence of rhizomorphs. However, *T. asperula* often has brownish olive hymenophoral surface and globose to subglobose basidiospores, which are distinctly different from *T. verruculata*.

## Discussion

In this study, six new *Tomentella* species from Vietnam are identified and described on the basis of morphological characteristics and phylogenetic analyses combining ITS and LSU sequences. The phylogenetic tree divided *Tomentella* into several distinct clades, and most of the clades are consistent with the previous ITS phylogenetic analyses with strong support or weak support (Yuan et al., 2020). Besides, some other clades with strong support are different from the previous study (Yuan et al., 2020). However, the results revealed a weak support at the deeper nodes of the tree, which is consistent with the previous studies (Kõljalg et al., 2000; Yorou et al., 2012b; Kuhar et al., 2016), and more taxa/genes are presumably needed to improve the stability of the tree. In addition, as the ITS region is very divergent within *Tomentella* species, it may be hard to get strong support for the internal clades.

*Tomentella cinereobrunnea* and *T.* sp. (TU115879, ECM) formed a strongly supported group (100% in ML and 1.00 BPP) according to the phylogenetic analyses, which indicate that *T.* sp. may belong to *T. cinereobrunnea*. The phylogenetic analyses also shows that *T. cinereobrunnea* linked to *T. lapida* without support, but they share lots of similar morphological and anatomical characteristics: basidiospores of approximately the same shape and size, the encrusted and clamped generative hyphae, and the absence of rhizomorphs and cystidia. However, *T. lapida* differ from *T. cinereobrunnea* by the basidiocarps adherent from the substrate, short and inflated subhymenial hyphal cells and the amyloid ornamentation of the basidiospores (Kõljalg, 1996). In this tree, *T. badia*, *T. longiechinula*, *T. cinereoumbrina*, and *T.* sp. (H1_7) from Asia and Europe are also closely related to *Thelephora atra* (synonym = *T. atramentaria* Rostr.) with significant support (90% in ML and 1.00 BPP). *T. cinereoumbrina* and *Th. atra* differ from *T. badia* and *T. longiechinula* by the crustose basidiocarps and *Th. atra* can be distinguished by its clamped hyphae (Kõljalg, 1996). Although species of the genus *Thelephora* and *Tomentella* do not separate to two monophyletic groups and they always form a well-supported clade in a phylogenetic tree (Tedersoo et al., 2014; Vizzini et al., 2016), the morphological and anatomical characteristics can set them apart from each other. In addition, simple-septate hyphae and basidiospores with warts are typical features that distinguish *T. bidoupensis* and *T. verruculata* from other species in *Tomentella* (Yuan et al., 2020).

The phylogenetic tree reveals that individual species of *Tomentella* can form ectomycorrhiza with different host tree species in different families, and closely related species in the same clade can be restricted to the same host tree family. In addition, the investigated forests were dominated by the coniferous trees *P. kesiya* mixed with families such as Ericaceae, Fagaceae, Lentibulariaceae, Orchidaceae, and Rosaceae (Nguyen et al., 2012; Pócs et al., 2019). We speculate that the plant hosts for these six new species of *Tomentella* are *P. kesiya* in Pinaceae or some species in Fagaceae according to the relationship showed in the phylogenetic tree and the dominated tree species in the investigated forests.

## Data Availability Statement

The datasets presented in this study can be found in online repositories. The names of the repository/repositories and accession number(s) can be found in the article/Supplementary Material.

## Author Contributions

H-SY conceived the study and gave the final approval of the manuscript to be published. H-SY and TN performed the investigation and sample the collection. XL and TC conducted the experiments and analyzed the data. XL wrote the original draft. All authors revised, read, and approved the final manuscript.

## Conflict of Interest

The authors declare that the research was conducted in the absence of any commercial or financial relationships that could be construed as a potential conflict of interest.

## Publisher’s Note

All claims expressed in this article are solely those of the authors and do not necessarily represent those of their affiliated organizations, or those of the publisher, the editors and the reviewers. Any product that may be evaluated in this article, or claim that may be made by its manufacturer, is not guaranteed or endorsed by the publisher.

## References

[B1] Agerer Agerer R. (1987-2008). *Colour Atlas of Ectomycorrhizas. 1st–14th Delivery.* Schwäbisch Gmünd: Einhorn-Verlag.

[B2] Agerer R. Bougher N. L. (2001). *Tomentella subamyloidea* sp. nov. and *T. radiosa* (Thelephoraceae, Hymenomycetes, Basidiomycota) from Australia. *Aust. Syst. Bot.* 14 607–614. 10.1071/SB00031

[B3] Alvarez-Manjarrez J. Garibay-Orijel R. Smith M. E. (2018). Caryophyllales are the main hosts of a unique set of ectomycorrhizal fungi in a neotropical dry forest. *Mycorrhiza* 28 103–115. 10.1007/s00572-017-0807-7 29181635

[B4] Benson D. A. Cavanaugh M. Clark K. Karsch-Mizrachi I. Ostell J. Pruitt K. D. (2018). GenBank. *Nucleic Acids Res.* 46 D41–D47. 10.1093/nar/gkx1094 29140468 PMC5753231

[B5] Cunningham G. H. (1963). *The Thelephoraceae of Australia and New Zealand. Bulletin of the New Zealand Department of Scientific and Industrial Research.* Wellington: R.E. Owen Bulletin: Government Printer, 1–359.

[B6] Danielson R. M. Visser S. Parkinson D. (1983). Microbial activity and mycorrhizal potential of four overburden types used in the reclamation of extracted oil sands. *Can. J. Soil. Sci.* 63 363–375. 10.4141/cjss83-035

[B7] Darriba D. Taboada G. L. Doallo R. Posada D. (2012). jModelTest 2: more models, new heuristics and parallel computing. *Nat. Methods* 9:772. 10.1038/nmeth.2109 22847109 PMC4594756

[B8] Disyatat N. R. Yomyart S. Sihanonth P. Piapukiew J. (2016). Community structure and dynamics of ectomycorrhizal fungi in a dipterocarp forest fragment and plantation in Thailand. *Plant Ecol. Divers.* 9 577–588. 10.1080/17550874.2016.1264018

[B9] Eskov A. K. Onipchenko V. G. Prilepsky N. G. Abakumov E. V. Kolomeitseva G. L. Thinh N. V. (2019). Dependence of epiphytic community on autochthonous and allochthonous sources of nitrogen in three forest habitats of southern Vietnam. *Plant Soil* 443 565–574. 10.1007/s11104-019-04252-1

[B10] Gao F. L. Chen C. J. Arab D. A. Du Z. G. He Y. H. Ho S. Y. W. (2019). EasyCodeML: a visual tool for analysis of selection using CodeML. *Ecol. Evol.* 9 1–8. 10.1002/ece3.5015 31015974 PMC6467853

[B11] Ge Z. W. Smith M. E. Zhang Q. Y. Yang Z. L. (2012). Two species of the Asian endemic genus *Keteleeria* form ectomycorrhizas with diverse fungal symbionts in Southwestern China. *Mycorrhiza* 22 403–408. 10.1007/s00572-011-0411-1 21997220

[B12] Huang J. Nara K. Zong K. Wang J. Xue S. Peng K. (2014). Ectomycorrhizal fungal communities associated with masson pine (*Pinus massoniana*) and white oak (*Quercus fabri*) in a manganese mining region in Hunan Province. *China. Fungal Ecol.* 9 1–10. 10.1016/j.funeco.2014.01.001

[B13] Ingleby K. Thuy L. T. T. Phong N. T. Mason P. A. (2000). Ectomycorrhizal inoculum potential of soils from forest restoration sites in South Vietnam. *J. Trop. For. Sci.* 12 418–422.

[B14] Jakucs E. Erős-Honti Z. Seress D. Kovács G. M. (2015). Enhancing our understanding of anatomical diversity in *Tomentella* ectomycorrhizas: characterization of six new morphotypes. *Mcorhiza* 25 419–429. 10.1007/s00572-014-0622-3 25564437

[B15] Kaewgrajang T. Sangwanit U. Kodama M. Yamato M. (2014). Ectomycorrhizal fungal communities of *Dipterocarpus alatus* seedlings introduced by soil inocula from a natural forest and a plantation. *J. For. Res.* 19 260–267. 10.1007/s10310-013-0408-z

[B16] Kõljalg U. (1996). *Tomentella (Basidiomycota) and Related Genera in Temperate Eurasia. Synopsis Fungorum.* Oslo: Fungiflora, 1–213.

[B17] Kõljalg U. Dahlberg A. Taylor A. F. S. Larsson E. Hallenberg N. Stenlid J. (2000). Diversity and abundance of resupinate thelephoroid fungi as ectomycorrhizal symbionts in Swedish boreal forests. *Mol. Ecol.* 9 1985–1996. 10.1046/j.1365-294X.2000.01105.x 11123611

[B18] Kornerup A. Wanscher J. (1981). *Methuen Handbook of Colour Fletcher.* Norwich: Fletcher & Son, 1–252.

[B19] Kuhar F. Barroetaveña C. Rajchenberg M. (2016). New species of *Tomentella* (Thelephorales) from the Patagonian Andes forests. *Mycologia* 108 780–790. 10.3852/15-244 27091385

[B20] Larsen M. J. (1974). *A contribution to the Taxonomy of the Genus Tomentella*. *Myco. Memoir.* Bronx, NY: New York Botanical Garden, 1–145.

[B21] Larsson K. H. Larsson E. Kõljalg U. (2004). High phylogenetic diversity among corticioid homobasidiomycetes. *Mycol. Res.* 108 983–1002. 10.1017/S0953756204000851 15506012

[B22] Larsson K. H. Svantesson S. Miscevic D. Kõljalg U. Larsson E. (2019). Reassessment of the generic limits for *Hydnellum* and *Sarcodon* (Thelephorales, Basidiomycota). *MycoKeys* 54 31–47. 10.3897/mycokeys.54.35386 31231164 PMC6579789

[B23] Li K. N. Rouse D. I. German T. L. (1994). PCR primers that allow intergeneric differentiation of ascomycetes and their application to *Verticillium* spp. *Appl. Environ. Microb.* 60 4324–4331. 10.1128/AEM.60.12.4324-4331 PMC201988 7811072

[B24] Lu X. Yuan H. S. (2021). New Species of *Tomentella* (Thelephorales, Basidiomycota) from Temperate Continental Mountain Climate of China (Xinjiang Region). *Forests* 12:1531. 10.3390/f12111531

[B25] Lu X. Mu Y. H. Yuan H. S. (2018a). Two new species of *Tomentella* (Thelephorales, Basidiomycota) from Lesser Xingan Mts., northeastern China. *Phytotaxa* 369 080–092. 10.11646/phytotaxa.369.2.2

[B26] Lu X. Steffen K. Yuan H. S. (2018b). Morphological and molecular identification of three new species of *Tomentella* from Finland. *Mycologia* 110 1–15. 10.1080/00275514.2018.1474683 30081774

[B27] Malysheva E. F. Malysheva V. F. Voronina E. Y. Kovalenko E. A. (2018). Diversity of fungal communities associated with mixotrophic pyroloids (*Pyrola rotundifolia*, *P. media* and *Orthilia secunda*) in their natural habitats. *Bot. Paci.* 7 31–39. 10.1007/s11118-011-9244-y

[B28] Moncalvo J. M. Rehner S. A. Vilgalys R. (1993). Systematics of *Lyophyllum* section *Difformia* based on evidence from culture studies and ribosomal DNA sequences. *Mycologia* 85 788–794. 10.1080/00275514.1993.12026333

[B29] Morris M. H. Smith M. E. Rizzo D. M. Rejmánek M. Bledsoe C. S. (2008). Contrasting ectomycorrhizal fungal communities on the roots of co-occurring oaks (*Quercus* spp.) in a California woodland. *New Phytol.* 178 167–176. 10.1111/j.1469-8137.2007.02348.x 18194145

[B30] Natarajan K. Chandrashekara K. V. (1978). A new species of *Tomentella* from South India. *Mycologia* 70 1294–1297. 10.1080/00275514.1978.12020356

[B31] Nguyen V. V. Nguyen Q. H. Nguyen T. M. H. Jung S. W. Hwang J. M. Bae Y. J. (2012). Aquatic insect fauna of Bidoup-Nui Ba National Park in Lam Dong Province. Southern Vietnam. B. *Entomol. Res.* 28 29–34.

[B32] Nilsson R. H. Larsson K. Taylor A. F. S. Bengtsson-Palme J. Jeppesen T. S. Schigel D. (2019). The UNITE database for molecular identification of fungi: handling dark taxa and parallel taxonomic classifications. *Nucleic Acids Res.* 47 D259–D264. 10.1093/nar/gky1022 30371820 PMC6324048

[B33] Nilsson R. H. Tedersoo L. Abarenkov K. Ryberg M. Kristiansson E. Hartmann M. (2012). Five simple guidelines for establishing basic authenticity and reliability of newly generated fungal ITS sequences. *Mycokeys* 4 37–63. 10.3897/mycokeys.4.3606

[B34] Nouhra E. Pastor N. Becerra A. Areitio E. S. Geml J. (2015). Greenhouse Seedlings of *Alnus* Showed Low Host Intrageneric Specificity and a Strong Preference for Some *Tomentella* Ectomycorrhizal Associates. *Microb. Ecol.* 69 813–825. 10.1007/s00248-014-0522-2 25370884

[B35] Peintner U. Dämmrich F. (2012). *Tomentella alpina* and other tomentelloid taxa fruiting in a glacier valley. *Mycol. Prog.* 11 109–119. 10.1007/s11557-010-0734-x

[B36] Pócs T. Tram N. K. T. He Q. Katagiri T. Luong T. T. (2019). New records for the liverwort and hornwort flora of Vietnam, 1. *Acta Bot. Hung.* 61 397–413. 10.1556/034.61.2019.3-4.9

[B37] Põlme S. (2018). *Ticodendron incognitum* and *Neea pittieri* associated ectomycorrhizal fungi in Neotropical mountain forest. *Asian J. Mycol.* 1 137–145. 10.5943/ajom/1/1/11

[B38] Read D. J. Perez-Moreno J. (2003). Mycorrhizas and nutrient cycling in ecosystems – a journey towards relevance? *New Phytol.* 157 475–492. 10.1046/j.1469-8137.2003.00704.x 33873410

[B39] Richard F. Roy M. Shahin O. Sthultz C. Duchemin M. Joffre R. (2011). Ectomycorrhizal communities in a Mediterranean forest ecosystem dominated by *Quercus ilex*: seasonal dynamics and response to drought in the surface organic horizon. *Ann. For. Sci.* 68 57–68. 10.1007/s13595-010-0007-5

[B40] Ronquist F. Huelsenbeck J. P. (2003). MrBayes 3: bayesian phylogenetic inference under mixed models. *Bioinformatics* 19 1572–1574. 10.1093/bioinformatics/btg180 12912839

[B41] Salomón M. E. S. Barroetaveña C. Pildain M. B. Kuhar F. Rajchenberg M. (2017). *Tomentella* (Thelephorales, Basidiomycota) en bosques de Nothofagaceae de Patagonia, Argentina: micorrizas de nuevas especies. *Soc. Argent. Bot. B Soc. Argent. Bot.* 52 423–434. 10.31055/1851.2372.v52.n3.18023

[B42] Silvestro D. Michalak I. (2012). raxmlGUI: a graphical front-end for RAxML. *Org. Divers. Evol.* 12 335–337. 10.1007/s13127-011-0056-0

[B43] Sirikantaramas S. Sugioka N. Lee S. S. Mohamed L. A. Lee H. S. Szmidt A. E. (2003). Molecular identification of ectomycorrhizal fungi associated with Dipterocarpaceae. *Tropics* 13 69–77. 10.3759/tropics.13.69

[B44] Smith M. E. Douhan G. W. Rizzo D. M. (2007). Ectomycorrhizal community structure in a xeric *Quercus* woodland based on rDNA sequence analysis of sporocarps and pooled roots. *New Phytol.* 174 847–863. 10.1111/j.1469-8137.2007.02040.x 17504467

[B45] Smith M. E. Henkel T. W. Aime M. C. Fremier A. K. Vilgalys R. (2011). Ectomycorrhizal fungal diversity and community structure on three co-occurring leguminous canopy tree species in a Neotropical rainforest. *New Phytol.* 192 699–712. 10.1111/j.1469-8137.2011.03844.x 21883231

[B46] Suvi T. Tedersoo L. Abarenkov K. Beaver K. Gerlach J. Kõljalg U. (2010). Mycorrhizal symbionts of *Pisonia grandis* and *P. sechellarum* in Seychelles: identification of mycorrhizal fungi and description of new *Tomentella* species. *Mycologia* 102 522–533. 10.3852/09-147 20524585

[B47] Tamura K. Peterson D. Peterson N. Stecher G. Nei M. Kumar S. (2011). MEGA5: molecular evolutionary genetics analysis using maximum likelihood, evolutionary distance, and maximum parsimony methods. *Mol. Biol. Evol.* 28 2731–2739. 10.1093/molbev/msr121 21546353 PMC3203626

[B48] Taylor D. L. McCormick M. K. (2008). Internal transcribed spacer primers and sequences for improved characterization of basidiomycetous orchid mycorrhizas. *New Phytol.* 177 1020–1033. 10.1111/j.1469-8137.2007.02320.x 18086221

[B49] Tedersoo L. Harend H. Buegger F. Pritsch K. Saar I. Kõljalg U. (2014). Stable isotope analysis, field observations and synthesis experiments suggest that *Odontia* is a non-mycorrhizal sister genus of *Tomentella* and *Thelephora*. *Fungal Ecol.* 11 80–90. 10.1016/j.funeco.2014.04.006

[B50] Tedersoo L. Jairus T. Horton B. M. Abarenkov K. Suvi T. Saar I. (2008). Strong host preference of ectomycorrhizal fungi in a Tasmanian wet sclerophyll forest as revealed by DNA barcoding and taxon-specific primers. *New Phytol.* 180 479–490. 10.1111/j.1469-8137.2008.02561.x 18631297

[B51] Tedersoo L. Mett M. Ishida T. A. Bahram M. (2013). Phylogenetic relationships among host plants explain differences in fungal species richness and community composition in ectomycorrhizal symbiosis. *New phytol.* 199 822–831. 10.1111/nph.12328 23692134

[B52] Tedersoo L. Suvi T. Beaver K. Kõljalg U. (2007). Ectomycorrhizal fungi of the Seychelles: diversity patterns and host shifts from the native *Vateriopsis seychellarum* (Dipterocarpaceae) and *Intsia bijuga* (Caesalpiniaceae) to the introduced *Eucalyptus robusta* (Myrtaceae), but not *Pinus caribea* (Pinaceae). *New Phytol.* 175 321–333. 10.1111/j.1469-8137.2007.02104.x 17587380

[B53] Tedersoo L. Suvi T. Larsson E. Kõljalg U. (2006). Diversity and community structure of ectomycorrhizal fungi in a wooded meadow. *Mycol. Res.* 110 734–748. 10.1016/j.mycres.2006.04.007 16769208

[B54] Thind K. S. Rattan S. S. (1971). Thelephoraceae of India. IV. The genus *Tomentella*. *Indian Phytopathol.* 24 32–42.

[B55] Vilgalys R. Hester M. (1990). Rapid genetic identification and mapping of enzymatically amplified ribosomal DNA from several *Cryptococcus* species. *J. Bacteriol.* 172 4238–4246. 10.1128/jb.172.8.4238-4246.1990 2376561 PMC213247

[B56] Vizzini A. Angelini C. Losi C. Ercole E. (2016). Thelephora dominicana (Basidiomycota, Thelephorales), a new species from the Dominican Republic, and preliminary notes on thelephoroid genera. *Phytotaxa* 265 27–38. 10.11646/phytotaxa.265.1.2

[B57] Wakefield E. M. (1966). Some extra-european species of *Tomentella*. *Trans. Brit. Mycol. Soc.* 49 357–362. 10.1016/S0007-1536(66)80077-3

[B58] Wakefield E. M. (1969). Tomentelloideae in the British Isles. *Trans. Brit. Mycol. Soc.* 53 161–206. 10.1016/S0007-1536(69)80053-7

[B59] Wang Q. Gao C. Guo L. D. (2011). Ectomycorrhizae associated with *Castanopsis fargesii*(Fagaceae) in a subtropical forest. *China. Mycol. Prog.* 10 323–332. 10.1007/s11557-010-0705-2

[B60] Wang Q. He X. H. Guo L. D. (2012). Ectomycorrhizal fungus communities of *Quercus liaotungensis* Koidz of different ages in a northern China temperate forest. *Mycorrhiza* 22 461–470. 10.1007/s00572-011-0423-x 22138969

[B61] Welden A. L. (1968). West Indian species of dark-spored Thelephoraceae. *Sydowia* 22 269–273.

[B62] Yorou N. S. (2008). *Miscellaneous Contributions to the Anatomy and Molecular Systematic of Tropical African Resupinate Thelephorales.* Ph.D. thesis. Munich: University of Munich.

[B63] Yorou N. S. Agerer R. (2008). *Tomentella africana*, a new species from Benin (West Africa) identified by morphological and molecular data. *Mycologia* 100 68–80. 10.1080/15572536.2008.11832499 18488353

[B64] Yorou N. S. Agerer R. (2011a). Rhizomorphic resupinate Thelephorales (Agaricomycetes, Basidiomycota) from Italy. *Nova Hedwigia* 92 177–204. 10.1127/0029-5035/2011/0092-0177

[B65] Yorou N. S. Agerer R. (2011b). Non-rhizomorphic resupinate Thelephorales (Agaricomycetes, Basidiomycota) from Italy. *Nova Hedwigia* 92 391–424. 10.1127/0029-5035/2011/0092-0391

[B66] Yorou N. S. Diabaté M. Agerer R. (2012a). Phylogenetic placement and anatomical characterisation of two new West African *Tomentella* (Basidiomycota, Fungi) species. *Mycol. Prog.* 11 171–180. 10.1007/s11557-011-0739-0

[B67] Yorou N. S. Gardt S. Guissou M. L. Diabaté M. Agerer R. (2012b). Three new *Tomentella* species from West Africa identified by anatomical and molecular data. *Mycol. Prog.* 11 449–462. 10.1007/s11557-011-0760-3

[B68] Yorou N. S. Guelly A. K. Agerer R. (2011). Anatomical and ITS rDNA-based phylogenetic identification of two new West African resupinate thelephoroid species. *Mycoscience* 52 363–375. 10.1007/S10267-011-0117-4

[B69] Yorou N. S. Kõljalg U. Sinsin B. Agerer R. (2007). Studies in African thelephoroid fungi: 1. *Tomentella capitata* and *Tomentella brunneocystidia*, two new species from Benin (West Africa) with capitate cystidia. *Mycol. Prog.* 6 7–18. 10.1007/s11557-006-0519-4

[B70] Yuan H. S. Lu X. Dai Y. C. Kevin D. H. Kan Y. H. Kušan I. (2020). Fungal diversity notes 1277–1386: taxonomic and phylogenetic contributions to fungal taxa. *Fungal Divers.* 1 1–260. 10.1007/s13225-020-00461-7

[B71] Yuan Y. Wu F. Dai Y. C. Qin W. M. Yuan H. S. (2018). *Odontia aculeata* and *O. sparsa*, two new species of tomentelloid fungi (Thelephorales, Basidiomycota) from the secondary forests of northeast China. *Phytotaxa* 372 183–192. 10.11646/phytotaxa.372.3.1

