# Supplementary material for: Six New Species of Tomentella (Thelephorales, Basidiomycota) From Tropical Pine Forests in Central Vietnam
Source: Front Microbiol. 2022 Apr 25;13:864198. doi: 10.3389/fmicb.2022.864198 (PMC9082317; doi:10.3389/fmicb.2022.864198)
Supplement: Supplementary file 1 [file Table_1.pdf]

Table 1. GenBank/UNITE accession numbers, voucher numbers, substrates, localities and references for the specimens included in this study. The newly generated sequences are in bold.

Species	GenBank No./UNITE Database accession No.		Voucher number	Substrate	Locality	references
	ITS	LSU				
Odontia ferruginea Pers.	UDB000285	_	TAAM149492	_	Estonia	Yuan et al., 2018
O. ferruginea	UDB025793	_	TU110988	_	Estonia	Yuan et al., 2018
Th. anthocephala (Bull.) Fr.	UDB000213	UDB018693	TAAM165304	_	Sweden	Kõljalg et al., 2000
Th. anthocephala	_	KP454019	UBC F28410	_	Canada	GenBank Database
Th. atra Weinm.	UDB000235	UDB018697	TAAM149211	_	Russian	Kõljalg et al., 2000
Th. atra	UDB026547	_	L9379	_	Italy	UNITE Database
Th. caryophyllea (Schaeff.) Pers.	_	MK602776	ELarsson89-09	_	Sweden	Larsson et al., 2019
Th. caryophyllea	UDB000212	UDB018694	TAAM172626	_	Estonia	Tedersoo et al., 2014

Th. ellisii (Sacc.) Jülich & Stalpers	UDB011603	_	TU115347	_	Finland	UNITE Database
Th. ellisii	UDB000226	_	TU123494	_	Germany	UNITE Database
Th. wakefieldiae Zmitr., Shchepin, Volobuev & Myasnikov	UDB011601	_	TU115353	_	Finland	UNITE Database
Th. wakefieldiae	UDB011599	_	TU115350	_	Finland	UNITE Database
Tomentella africana Yorou & Agerer	EF507254	_	SYN 991	Undersides of dead, burned barks, logs, and leaf litter of native trees	Benin	Yorou and Agerer, 2008
T. africana	EF507256	_	SYN890	Undersides of dead, burned barks, logs, and leaf litter of native trees	Benin	Yorou and Agerer, 2008
T. afrostuposa Yorou	JF520431	_	SYN 2292	On dead valves of *Afzelia africana* Smith ex Pers.	Guinea	Yorou et al., 2012b
T. afrostuposa	NR119954	_	M SYN 2292	On dead valves of *A. africana*	Guinea	Yorou et al., 2012b
T. agbassaensis	EF507257	_	SYN 981	Undersides of dead, burned barks, logs, and leaf litter of native trees	Benin	Yorou et al., 2012a
T. agbassaensis Yorou	NR119638	_	M SYN 981	On dead valves of *A. africana*	Benin	Yorou et al., 2012a

T. agereri Yorou	EF538424	_	RA 13793	On dead valves of *A. africana*	Benin	Yorou et al., 2011
T. agereri	NR119641	_	M RA 13793	On dead valves of *A. africana*	Benin	Yorou et al., 2011
T. alpina Peintner & Dämmrich	EF655702	_	IB 20060231	ECM root tips of *Polygonum viviparum* L.	Austria	Peintner and Dämmrich, 2012
T. alpina	NR121330	_	B20060231	ECM root tips of *P. viviparum*	Austria	Peintner and Dämmrich, 2012
T. amyloapiculata Yorou	EF507263	_	M SYN 893	On dead valves of *A. africana*	Benin	Yorou et al., 2012a
T. amyloapiculata	_	UDB016726	TU102067	_	Zambia	UNITE Database
T. asiae-orientalis H.S. Yuan, X. Lu & Y.C. Dai	MK211711	MK446334	Yuan 12022	On fallen angiosperm twig	China	Yuan et al., 2020
T. asiae-orientalis	MK211710	MK446333	Yuan 11918	On fallen branch of *Pinus koraiensis*	China	Yuan et al., 2020
T. asperula (P. Karst.) Höhn. & Litsch.	_	UDB018469	TU108147	_	Estonia	UNITE Database
T. asperula	KF498576	_	MT7	*Fagus sylvatica* L.	Germany	NCBI Database
T. atroarenicolor Nikol.	_	UDB018480	TU100676	Under decayed wood	Estonia	UNITE Database

T. atroarenicolor	_	UDB016303	TU115438	_	Estonia	UNITE Database
T. atrobadia H.S. Yuan & Y.C. Dai	KY686248	MK446335	Yuan 11099	On rotten angiosperm branch	China	Yuan et al., 2020
T. atrobadia	KY686249	MK446336	Yuan 11114	On rotten angiosperm wood debris	China	Yuan et al., 2020
T. atrocastanea H.S. Yuan, X. Lu & Y.C. Dai	MK211743	MK446338	Yuan 12179	On rotten angiosperm wood debris	China	Yuan et al., 2020
T. atrocastanea	MK211742	MK446337	Yuan 12170	On rotten angiosperm wood debris	China	Yuan et al., 2020
T. aureomarginata H.S. Yuan, X. Lu & Y.C. Dai	MK211744	MK446339	Yuan 10671	On rotten angiosperm wood debris	China	Yuan et al., 2020
T. aureomarginata	MK211745	MK878395	Yuan 10683	On rotten angiosperm wood debris	China	Yuan et al., 2020
T. badia (Link) Stalpers	UDB000952	_	UK427	_	Estonia	UNITE Database
T. badia	UDB000238	_	TAA159022	_	Russia	Kõljalg et al., 2000
T. beaverae Suvi & Kõljalg	_	UDB015002	TU105060	*Intsia bijuga* (Colebr.) O. Kuntze	Seychelles	Tedersoo et al., 2007
T. beaverae	_	UDB017787	TU103595	*I. bijuga*	Seychelles	Tedersoo et al., 2007

T. bidoupensis	**MK775477**	**MN684329**	**Yuan 12707**	**On rotten wood debris of *Pinus kesiya***	**Vietnam**	**This study**
T. bidoupensis	**MK775476**	**MN684330**	**Yuan 12685**	**On rotten wood debris of *P. kesiya***	**Vietnam**	**This study**
T. botryoides (Schwein.) Bourdot & Galzin	UDB000255	AY586717	KHL8453	_	Sweden	Larsson et al., 2004
T. botryoides	UDB000257	_	TAAM149614	_	Russia	Kõljalg et al., 2000
T. bresadolae (Brink-mann) Bourdot & Gal-zin	UDB020335	_	TU115616	_	Slovenia	UNITE Data-base
T. bresadolae	UDB016311	_	TU115447	_	Estonia	UNITE Data-base
T. brevis H.S. Yuan, X. Lu & Y.C. Dai	MK211746	MK446340	Yuan 11328	On fallen angiosperm branch	China	Yuan et al., 2020
T. brevis	MK211747	MK878396	Yuan 11332	On fallen angiosperm branch	China	Yuan et al., 2020
T. brevisterigmata	**MK775472**	**MK850202**	**Yuan 12700**	**On rotten wood debris of *P. kesiya***	**Vietnam**	**This study**
T. brevisterigmata	**MK775473**	**MK850203**	**Yuan 12701**	**On rotten wood debris of *P. kesiya***	**Vietnam**	**This study**
T. brunneocystidia Yorou & Agerer	DQ848613	_	SYN 839 (M)	On dead barks and logs of native trees	Benin	Yorou et al., 2007

T. brunneocystidia	DQ848610	_	RA 13779	On dead barks and logs of native trees	Benin	Yorou et al., 2007
T. brunneoflava H.S. Yuan, X. Lu & Y.C. Dai	MK211749	MK850194	Yuan 12162	On rotten angiosperm wood debris	China	Yuan et al., 2020
T. brunneoflava	MK211748	MK446341	Yuan 12161	On fallen branch of *Larix* sp.	China	Yuan et al., 2020
T. brunneogrisea H.S. Yuan, X. Lu & Y.C. Dai	MK211751	MK446343	Yuan 12147	On fallen angiosperm branch	China	Yuan et al., 2020
T. brunneogrisea	MK211750	MK446342	Yuan 12146	On fallen angiosperm branch	China	Yuan et al., 2020
T. brunneorufa M.J. Larsen	UDB000274	_	TAAM159857	*Thelephora-Tomentella* EcM line-ages	Australia	UNITE Data-base
T. bryophila (Pers.) M.J. Larsen	UDB014252	_	TU116131	_	Estonia	UNITE Data-base
T. bryophila	_	UDB028250	TU124259	_	Estonia	UNITE Data-base
T. capitata Yorou & Agerer	DQ848611	_	RA13785 (M)	On dead bark and log	Benin	Yorou et al., 2007
T. capitata	DQ848612	_	SYN 860 (M)	On dead bark and log	Benin	Yorou et al., 2007
T. capitatocystidiata H.S. Yuan, X. Lu & Y.C. Dai	MK211700	MK446344	Yuan 11459	On fallen angiosperm branch	China	Yuan et al., 2020

T. capitatocystidiata	MK211701	MK446345	Yuan 11494	On fallen angiosperm branch	China	Yuan et al., 2020
T. castanea (Bourdot & Galzin) Donk	UDB005597	_	B923	_	Iran	UNITE Data-base
T. castanea	UDB000120	_	TL-6886	_	Denmark	Nouhra et al., 2015
T. changbaiensis H.S. Yuan, X. Lu & Y.C. Dai	MK211739	MK446347	Yuan 11496	On fallen angiosperm branch	China	Yuan et al., 2020
T. changbaiensis	MK211738	MK446346	Yuan 11477	On fallen angiosperm branch	China	Yuan et al., 2020
T. cinerascens (P. Karst.) Höhn. & Litsch.	_	UDB016193	TU108037	_	Estonia	UNITE Data-base
T. cinerascens	_	UDB016498	TU111378	_	Italy	UNITE Data-base
T. cinereobrunnea	**MK775478**	**MK850198**	**Yuan 12703**	**On rotten wood debris of *P. kesiya***	**Vietnam**	**This study**
T. cinereobrunnea	**MK775479**	**MK850199**	**Yuan 12705**	**On rotten wood debris of *P. kesiya***	**Vietnam**	**This study**
T. cinereoumbrina (Bres.) Stalpers	UDB011602	_	TU115342	_	Finland	UNITE Data-base
T. cinereoumbrina	UDB016491	_	TU111371	_	Italy	UNITE Data-base

T. citrinocystidiata H.S. Yuan & Y.C. Dai	KY686246	MK446348	Yuan 10680	On rotten angiosperm wood debris	China	Yuan et al., 2020
T. citrinocystidiata	KY686247	MK446349	Yuan 10743	On rotten angiosperm wood debris	China	Yuan et al., 2020
T. clavigera Litsch.	UDB016389	_	TU115532	_	Estonia	UNITE Database
T. coerulea Höhn. & Litsch.	UDB016469	_	TU115602	_	Estonia	UNITE Database
T. coerulea	UDB000266	_	TAAM153804	_	Estonia	UNITE Database
T. coffeae H.S. Yuan & Y.C. Dai	KY686254	MK446350	Yuan 10629	On rotten angiosperm wood debris	China	Yuan et al., 2020
T. coffeae	KY686255	MK446351	Yuan 11100	On fallen angiosperm branch	China	Yuan et al., 2020
T. conclusa H.S. Yuan, X. Lu & Y.C. Dai	MK211703	MK850195	Yuan 12086	On fallen angiosperm branch	China	Yuan et al., 2020
T. conclusa	MK211702	MK446352	Yuan 11986	On fallen trunk of *P. koraiensis*	China	Yuan et al., 2020
T. cystidiata H.S. Yuan & Y.C. Dai	KY686219	MK446353	Yuan 10620	On rotten angiosperm wood debris	China	Yuan et al., 2020
T. cystidiata	KY686218	MK446354	Yuan 10693	On rotten angiosperm wood debris	China	Yuan et al., 2020

T. dimidiata H.S. Yuan, X. Lu & Y.C. Dai	MK211704	MK446355	Yuan 11205	On fallen angiosperm branch	China	Yuan et al., 2020
T. dimidiata	MK211705	MK446356	Yuan 11267	On fallen angiosperm branch	China	Yuan et al., 2020
T. duplexa H.S. Yuan, X. Lu & Y.C. Dai	MK211707	MK446358	Yuan 12207	On rotten angiosperm wood debris	China	Yuan et al., 2020
T. duplexa	MK211706	MK446357	Yuan 12202	On rotten angiosperm wood debris	China	Yuan et al., 2020
T. efibulata H.S. Yuan & Y.C. Dai	KY686228	MK446359	Yuan 10699	On rotten angiosperm wood debris	China	Yuan et al., 2020
T. efibulata	KY686229	MK446360	Yuan 11167	On rotten angiosperm wood debris	China	Yuan et al., 2020
T. efibulis H.S. Yuan, X. Lu & Y.C. Dai	MK211708	MK446361	Yuan 11241	On fallen angiosperm branch	China	Yuan et al., 2020
T. efibulis	MK211709	MK446362	Yuan 11329	On fallen angiosperm branch	China	Yuan et al., 2020
T. farinosa H.S. Yuan & Y.C. Dai	KY686251	_	Yuan 10656	On rotten angiosperm wood debris	China	Yuan et al., 2020
T. farinosa	KY686250	_	Yuan 10666	On rotten angiosperm trunk	China	Yuan et al., 2020
T. flavidobadia H.S. Yuan & Y.C. Dai	KY686231	MK446364	Yuan 11044	On fallen angiosperm branch	China	Yuan et al., 2020

T. flavidobadia	KY686230	MK446365	Yuan 11061	On fallen angiosperm branch	China	Yuan et al., 2020
T. fuscocinerea (Pers.) Donk	UDB000960	_	KHL11906	_	Sweden	UNITE Data-base
T. fuscocinerea	UDB000240	UDB018703	TAAM149918	_	Estonia	Kõljalg et al., 2000
T. fuscocrustosa H.S. Yuan, X. Lu & Y.C. Dai	MK211713	MK446367	Yuan 11420	On fallen angiosperm branch	China	Yuan et al., 2020
T. fuscocrustosa	MK211712	MK446366	Yuan 11399	On fallen angiosperm branch	China	Yuan et al., 2020
T. fuscofarinosa H.S. Yuan, X. Lu & Y.C. Dai	MK211715	MK446369	Yuan 12142	On fallen angiosperm branch	China	Yuan et al., 2020
T. fuscofarinosa	MK211714	MK446368	Yuan 12125	On fallen angiosperm branch	China	Yuan et al., 2020
T. fuscogranulosa H.S. Yuan & Y.C. Dai	KY686232	MK446370	Yuan 10733	On fallen angiosperm branch	China	Yuan et al., 2020
T. fuscogranulosa	KY686233	MK446371	Yuan 10725	On fallen angiosperm branch	China	Yuan et al., 2020
T. fuscopelliculosa H.S. Yuan, X. Lu & Y.C. Dai	MK211716	MK446372	Yuan 11305	On fallen angiosperm branch	China	Yuan et al., 2020
T. fuscopelliculosa	MK211717	MK446373	Yuan 11316	On fallen angiosperm branch	China	Yuan et al., 2020

T. galzinii Bourdot	UDB000264	_	RS27093	_	Finland	UNITE Data-base
T. galzinii	HQ204743	_	2007BBF2	*Quercus ilex* L.	France	Richard et al., 2011
T. globosa X. Lu, K. Steffen & H.S. Yuan	MG136838	MH201367	Yuan 11618	On rotten angiosperm wood debris	Finland	Lu et al., 2018
T. globosa	MG136839	MN684328	Yuan 11603	On rotten angiosperm wood debris	Finland	Lu et al., 2018
T. globospora H.S. Yuan & Y.C. Dai	KY686242	MK446374	Yuan 10668	On rotten angiosperm wood debris	China	Yuan et al., 2020
T. globospora	KY686243	MK446375	Yuan 10748	On rotten angiosperm wood debris	China	Yuan et al., 2020
T. gloeocystidiata H.S. Yuan & Y.C. Dai	KY686220	MK446376	Yuan 11171	On rotten angiosperm wood debris	China	Yuan et al., 2020
T. gloeocystidiata	KY686221	MK446377	Yuan 11200	On rotten angiosperm wood debris	China	Yuan et al., 2020
T. griseocastanea H.S. Yuan, X. Lu & Y.C. Dai	MK211719	MK446379	Yuan 11409	On fallen angiosperm branch	China	Yuan et al., 2020
T. griseocastanea	MK211718	MK446378	Yuan 11401	On fallen angiosperm branch	China	Yuan et al., 2020
T. griseofusca H.S. Yuan & Y.C. Dai	KY686252	MK446380	Yuan 11094	On rotten angiosperm branch	China	Yuan et al., 2020

T. griseofusca	KY686253	MK446381	Yuan 11104	On rotten angiosperm wood debris	China	Yuan et al., 2020
T. griseomarginata H.S. Yuan, X. Lu & Y.C. Dai	MK211721	MK446383	Yuan 11468	On fallen angiosperm branch	China	Yuan et al., 2020
T. griseomarginata	MK211720	MK446382	Yuan 11458	On fallen angiosperm branch	China	Yuan et al., 2020
T. guineensis Yorou	JF520432	_	SYN 2331	On dead logs, under *A. africana*	Guinea	Yorou et al., 2012b
T. guineensis	NR119955	_	M SYN 2331	On dead valves of *A. africana*	Guinea	Yorou et al., 2012b
T. hjortstamiana Suvi & Kõljalg	AM412303	_	TU103641	*I. bijuga*	Seychelles	Suvi et al., 2010
T. hjortstamiana	KC222770	_	Toohyp24	_	Australia	NCBI Data-base
T. inconspicua H.S. Yuan & Y.C. Dai	KY686234	MK446385	Yuan 11107	On rotten angiosperm branch	China	Yuan et al., 2020
T. inconspicua	KY686235	MK446384	Yuan 11060	On rotten angiosperm wood debris	China	Yuan et al., 2020
T. incrustata H.S. Yu-an, X. Lu & Y.C. Dai	MK211723	MK446387	Yuan 12189	On fallen angiosperm branch	China	Yuan et al., 2020
T. incrustata	MK211722	MK446386	Yuan 11158	On fallen angiosperm branch	China	Yuan et al., 2020

T. interrupta H.S. Yuan & Y.C. Dai	KY686236	MK446388	Yuan 10775	On rotten angiosperm wood debris	China	Yuan et al., 2020
T. interrupta	KY686237	MK446389	Yuan 11203	On rotten angiosperm branch	China	Yuan et al., 2020
T. intsiae Suvi & Kõljalg	UDB039732	_	TU123956	_	Seychelles	UNITE Database
T. intsiae	AM412296	_	TU105130	*I. bijuga*	Seychelles	Suvi et al., 2010
*T. lammiensis*X. Lu, K. Steffen & H.S. Yuan	MG136840	MH201366	Yuan 11617	On rotten angiosperm wood debris	Finland	Lu et al., 2018
T. lammiensis	MG136841	MH201364	Yuan 11597	On rotten angiosperm wood debris and broad leaf litter	Finland	Lu et al., 2018
T. lapida (Pers.) Stalpers	_	UDB016370	TU115604	_	Estonia	UNITE Database
T. lapida	_	UDB016305	TU115440	_	Estonia	UNITE Database
T. larssoniana Suvi & Kõljalg	_	UDB017785	TU103690	*I. bijuga*	Seychelles	Tedersoo et al., 2007
T. larssoniana	UDB017790	_	TU105082	*I. bijuga*	Seychelles	Suvi et al., 2010
T. lateritia Pat.	UDB000963	_	NF S045	_	Norway	UNITE Database

T. lateritia	UDB000954	_	TU108551	_	Estonia	UNITE Data-base
T. liaoningensis H.S. Yuan & Y.C. Dai	MK250814	_	Yuan 10681	On rotten angiosperm wood debris	China	Yuan et al., 2020
T. liaoningensis	KY686257	_	Yuan 10707	On fallen angiosperm branch	China	Yuan et al., 2020
T. lilacinogrisea Wakef.	_	UDB018468	TU108189	_	Estonia	UNITE Data-base
T. lilacinogrisea	_	UDB016500	TU111381	_	Italy	UNITE Data-base
T. longiaculeifera H.S. Yuan & Y.C. Dai	KY686238	MK446391	Yuan 10744	On bark of fallen angiosperm trunk	China	Yuan et al., 2020
T. longiaculeifera	KY686239	MK446392	Yuan 11119	On rotten angiosperm branch	China	Yuan et al., 2020
T. longiechinula	**MK775474**	**MK850201**	**Yuan 12687**	**On rotten wood debris of *P. kesiya***	**Vietnam**	**This study**
T. longiechinula	**MK775475**	**MK850200**	**Yuan 12720**	**On rotten wood debris of *P. kesiya***	**Vietnam**	**This study**
T. longiechinuli H.S. Yuan, X. Lu & Y.C. Dai	MK211726	MK446393	Yuan 11979	On fallen angiosperm branch	China	Yuan et al., 2020
T. longiechinuli	MK211727	MK446394	Yuan 12083	On fallen angiosperm branch	China	Yuan et al., 2020

T. longisterigmata X. Lu, K. Steffen & H.S. Yuan	MG136836	MN684325	Yuan 11610	On rotten angiosperm wood debris.	Finland	Lu et al., 2018
T. longisterigmata	MG136837	MH201365	Yuan 11602	On rotten angiosperm wood debris.	Finland	Lu et al., 2018
T. maroana Yorou	EF507250	_	SYN 878	Undersides of dead, burned barks, logs, and leaf litter of native trees.	Benin	Yorou and Agerer, 2008
T. maroana	NR119636	_	M SYN 878	Undersides of dead, burned barks, logs, and leaf litter of native trees.	Benin	Yorou and Agerer, 2008
T. megaspora H.S. Yuan, X. Lu & Y.C. Dai	MK211724	MK446395	Yuan 11326	On fallen angiosperm branch	China	Yuan et al., 2020
T. megaspora	MK211725	MK446396	Yuan 11472	On fallen angiosperm branch	China	Yuan et al., 2020
T. muricata (Ellis & Everh.) Wakef.	UDB003303	_	TU100771	_	Estonia	UNITE Database
T. muricata	UDB003310	_	TU100729	_	Finland	UNITE Database
T. nitellina Bourdot & Galzin	EF411085	_	L2AA1	*Quercus wislizeni* A. DC.	USA	Morris et al., 2008
T. nitellina	DQ974778	_	src675	*Quercus douglasii* Hook. & Arn.	USA	Smith et al., 2007
T. olivacea H.S. Yuan & Y.C. Dai	KY686224	MK446397	Yuan 11043	On rotten angiosperm branch	China	Yuan et al., 2020

T. olivacea	KY686225	MK446398	Yuan 11139	On rotten angiosperm branch	China	Yuan et al., 2020
T. olivaceobrunnea H.S. Yuan, X. Lu & Y.C. Dai	MK211728	MK446399	Yuan 11194	On rotten angiosperm wood debris	China	Yuan et al., 2020
T. olivaceobrunnea	MK211729	MK446400	Yuan 12148	On rotten angiosperm wood debris	China	Yuan et al., 2020
T. pallidobrunnea H.S. Yuan, X. Lu & Y.C. Dai	MK211731	MK446402	Yuan 11493	On rotten angiosperm branch	China	Yuan et al., 2020
T. pallidobrunnea	MK211730	MK446401	Yuan 11481	On fallen angiosperm branch	China	Yuan et al., 2020
T. pallidocastanea X. Lu, Y.H. Mu & H.S. Yuan	MG799183	MN684323	Yuan 11416	On rotten angiosperm wood debris	China	Lu et al., 2018
T. pallidocastanea	MG816514	MN684324	Yuan 12034	On rotten angiosperm wood debris	China	Lu et al., 2018
T. pallidomarginata H.S. Yuan, X. Lu & Y.C. Dai	MK211733	MK446404	Yuan 11474	On fallen angiosperm branch	China	Yuan et al., 2020
T. pallidomarginata	MK211732	MK446403	Yuan 11404	On fallen angiosperm branch	China	Yuan et al., 2020
T. parmastoana Suvi & Kõljalg	_	UDB016713	TU105091	*I. bijuga*	Seychelles	Suvi et al., 2010
T. parmastoana	_	UDB017782	TU103691	*I. bijuga*	Seychelles	Suvi et al., 2010

T. parvispora H.S. Yuan & Y.C. Dai	KY686226	MK446405	Yuan 11144	On fallen angiosperm branch	China	Yuan et al., 2020
T. parvispora	KY686227	MK446406	Yuan 11196	On fallen angiosperm branch	China	Yuan et al., 2020
T. patagonica Kuhar & Rajchenb.	KT032091	KT032103	BAFC52373	On rotten wood under *Nothofagus dombeyi* Mirb.	Argentina	Kuhar et al., 2016
T. patagonica	KT032090	KT032102	BAFC52372	On rotten wood under *N. dombeyi*	Argentina	Kuhar et al., 2016
T. pertenuis H.S. Yuan & Y.C. Dai	KY686240	MK446407	Yuan 11064	On rotten angiosperm branch	China	Yuan et al., 2020
T. pertenuis	KY686241	MK446408	Yuan 11131	On rotten angiosperm stump	China	Yuan et al., 2020
T. pileocystidiata Suvi & Kõljalg	UDB015029	_	TU105068	*I. bijuga*	Seychelles	Suvi et al., 2010
T. pileocystidiata	UDB017789	_	TU105054	*I. bijuga*	Seychelles	Suvi et al., 2010
T. pilosa (Burt) Bourdot & Galzin	_	UDB028059	TU124067	_	Estonia	UNITE Database
T. pilosa	_	UDB028227	TU124234	_	Estonia	UNITE Database
T. pisoniae Suvi & Kõljalg	_	UDB002643	TU103671	*Pisonia grandis* R. Br	Seychelles	Suvi et al., 2010

T. pisoniae	UDB017778	_	TU103655	*P. grandis*	Seychelles	Suvi et al., 2010
T. pulvinulata Kuhar & Rajchenb	KT032089	_	BAFC52371	On rotten wood under *N. dombeyi*	Argentina	Kuhar et al., 2016
T. pulvinulata	KT032088	KT032101	BAFC52370	On rotten wood under *N. dombeyi*	Argentina	Kuhar et al., 2016
T. punicea (Alb. & Schwein.) J. Schröt.	_	UDB008231	TU110254	_	Estonia	UNITE Database
T. punicea	UDB000959	_	KHL11908	_	Sweden	Tedersoo et al., 2006
T. pyrolae (Ellis & Halst.) M.J. Larsen	UDB000262	_	TAAM005998	_	Switzerland	UNITE Database
T. qingyuanensis H.S. Yuan & Y.C. Dai	KY686223	MK446409	Yuan 10616	On rotten angiosperm branch	China	Yuan et al., 2020
T. qingyuanensis	KY686222	MK446410	Yuan 11109	On rotten angiosperm wood debris	China	Yuan et al., 2020
T. radiosa (P. Karst.) Rick	_	UDB014068	TU110022	_	Ecuador	UNITE Database
T. radiosa	UDB000964	_	NF.S010	_	Norway	UNITE Database
T. segregata H.S. Yuan, X. Lu & Y.C. Dai	MK211735	MK446412	Yuan 11256	On fallen angiosperm branch	China	Yuan et al., 2020

T. segregata	MK211734	MK446411	Yuan 10650	On living tree root	China	Yuan et al., 2020
T. separata H.S. Yuan, X. Lu & Y.C. Dai	MK211737	MK850196	Yuan 10664	On fallen angiosperm trunk	China	Yuan et al., 2020
T. separata	MK211736	MK850197	Yuan 10654	On fallen angiosperm branch	China	Yuan et al., 2020
T. stipitata H.S. Yuan, X. Lu & Y.C. Dai	MK211740	MK446413	Yuan 11160	On fallen angiosperm branch	China	Yuan et al., 2020
T. stipitata	MK211741	MK446414	Yuan 12143	On fallen angiosperm branch	China	Yuan et al., 2020
T. stipitobasidia	**MK775470**	**MK850204**	**Yuan 12713**	**On wood debris of *P. kesiya***	**Vietnam**	**This study**
T. stipitobasidia	**MK775471**	**MK850205**	**Yuan 12691**	**On wood debris of *P. kesiya***	**Vietnam**	**This study**
T. storea H.S. Yuan & Y.C. Dai	KY686244	MK446415	Yuan 10623	On rotten angiosperm wood debris	China	Yuan et al., 2020
T. storea	KY686245	MK446416	Yuan 10749	On rotten angiosperm wood debris	China	Yuan et al., 2020
T. stuposa (Link) Stalpers	–	MK602778	Th0764	–	Norway	Larsson et al., 2019
T. stuposa	_	UDB016174	TU115328	_	Estonia	UNITE Data-base

T. subclavigera Litsch.	_	UDB031979	TU115594	_	Finland	UNITE Database
T. subclavigera	_	UDB031983	TU115593	_	Finland	UNITE Database
T. subtestacea Bourdot & Galzin	_	UDB016180	TU115374	_	Ukraine	UNITE Database
T. subtestacea	_	UDB016340	TU115482	_	Estonia	UNITE Database
T. tedersooi Suvi & Kõljalg	UDB017781	_	TU103673	*P. grandis*	Seychelles	Suvi et al., 2010
T. tedersooi	UDB002644	_	TU103664	*P. grandis*	Seychelles	Suvi et al., 2010
T. tenuirhizomorpha X. Lu, Y.H. Mu & H.S. Yuan	MG799184	MN684326	Yuan 11964	On rotten angiosperm wood debris	China	Lu et al., 2018
T. tenuirhizomorpha	MG799185	MN684327	Yuan 12059	On rotten angiosperm wood debris	China	Lu et al., 2018
T. tenuissima Kuhar & Rajchenb	KT032083	_	FK15011	On rotten wood under *Nothofagus pumilio* (Poepp. et Endl.) Krasser	Argentina	Kuhar et al., 2016
T. tenuissima	KT032082	KT032100	BAFC52369	Under a cushion of mosses in a pure *N. pumilio* forest	Argentina	Kuhar et al., 2016
T. terrestris (Berk. & Broome) M.J. Larsen	UDB000222	UDB018708	EL9897	_	USA	Kõljalg et al., 2000

T. terrestris	UDB003315	_	TU100886	_	France	UNITE Data-base
T. umbrinospora M.J. Larsen	–	UDB016499	TU111379	_	Italy	UNITE Data-base
T. umbrinospora	UDB000233	UDB018709	TAAM149462	_	Estonia	Tedersoo et al., 2014
T. verruculata	**MK775469**	**MN684332**	**Yuan 12684**	**On wood debris of *P. kesiya***	**Vietnam**	**This study**
T. verruculata	**MK775468**	**MN684331**	**Yuan 12680**	**On wood debris of *P. kesiya***	**Vietnam**	**This study**
T. viridula (Bourdot & Galzin) Svrček	–	UDB016192	TU108038	_	Estonia	UNITE Data-base
T. viridula	UDB016392	_	TU115536	_	Estonia	UNITE Data-base
T. sp. (SH1893901.08FU)	–	UDB018438	TU115005	*Thelephora*-*Tomentella* EcM lineages	China	Tedersoo et al., 2014
T. sp. (SH1893919.08FU)	–	UDB018441	TU115009	*Thelephora*-*Tomentella* EcM lineages	China	Tedersoo et al., 2014
T. sp. (SH1854116.08FU)	–	UDB018445	TU115018	*Thelephora*-*Tomentella* EcM lineages	China	Tedersoo et al., 2014
T. sp. (SH1893873.08FU)	–	UDB018446	TU115020	*Thelephora*-*Tomentella* EcM lineages	China	Tedersoo et al., 2014
T. sp. (SH1854194.08FU)	–	UDB018447	TU115022	*Thelephora*-*Tomentella* EcM lineages	China	Tedersoo et al., 2014

T. sp. (SH1854043.08FU)	–	UDB018448	TU115024	*Thelephora-Tomentella* EcM lineages	China	Tedersoo et al., 2014
T. sp. (SH1894205.08FU)	–	UDB018449	TU115027	*Thelephora-Tomentella* EcM lineages	China	Tedersoo et al., 2014
T. sp. (SH1855085.08FU)	–	UDB018451	TU115032	*Thelephora-Tomentella* EcM lineages	China	Tedersoo et al., 2014
T. sp. (SH1853609.08FU)	–	UDB018452	TU115035	*Thelephora-Tomentella* EcM lineages	China	Tedersoo et al., 2014
T. sp. (SH1854117.08FU)	–	UDB018453	TU115037	*Thelephora-Tomentella* EcM lineages	China	Tedersoo et al., 2014
T. sp. (SH1889504.08FU)	–	UDB018456	TU115042	*Thelephora-Tomentella* EcM lineages	China	Tedersoo et al., 2014
T. sp. (SH1853832.08FU)	UDB018460	_	TU115046	*Thelephora-Tomentella* EcM lineages	China	Tedersoo et al., 2014
T. sp. (SH1854133.08FU)	JN129412	_	H1_7	*Keteleeria davidiana* var. *calcerea*	China	Ge et al., 2012
T. sp. (SH1642921.08FU)	GQ900536	_	ECM6	*Castanopsis fargesii*	China	Wang et al., 2011
T. sp. (SH1894287.08FU)	GQ900538	_	ECM4	*C. fargesii*	China	Wang et al., 2011
T. sp. (SH1642921.08FU)	AB769926	_	94831	*Pinus massoniana*	China	Huang et al., 2014
T. sp. (SH1853124.08FU)	AB769927	_	P51	*P. massoniana*	China	Huang et al., 2014
T. sp. (SH1894152.08FU)	HM105509	_	QL011	*Quercus liaotungensis*	China	Huang et al., 2014
T. sp. (SH1855032.08FU)	HM105528	_	QL034	*Q. liaotungensis*	China	Wang et al., 2012
T. sp. (SH1894374.08FU)	HM105550	_	QL075	*Q. liaotungensis*	China	Wang et al., 2012

T. sp. (SH1853478.08FU)	HM105555	–	QL084	*Q. liaotungensis*	China	Wang et al., 2012
T. sp. (SH1894204.08FU)	HM105561	–	QL095	*Q. liaotungensis*	China	Wang et al., 2012
T. sp. (SH accession no. unavailable)	AB453038	–	CU:Micro:Nan-Morphotype Tomen 1	Dipterocarpaceae	Thailand	Disyatat et al., 2016
T. sp. (SH accession no. unavailable)	AB453039	–	CU:Micro:Nan-Morphotype Tomen 2	Dipterocarpaceae	Thailand	Disyatat et al., 2016
T. sp. (SH1894344.08FU)	AB777488	–	CP149	*Dipterocarpus alatus*	Thailand	Kaewgrajang et al., 2014
T. sp. (SH1854998.08FU)	AB777491	–	CP333	*D. alatus*	Thailand	Kaewgrajang et al., 2014
T. sp. (SH1853956.08FU)	UDB025279	–	TU116518	*Thelephora-Tomentella* EcM lineages	Thailand	UNITE Database
T. sp. (SH1854181.08FU)	UDB025280	–	TU116519	–	Thailand	UNITE Database
T. sp. (SH1502450.08FU)	UDB025283	–	TU116522b	*Thelephora-Tomentella* EcM lineages	Thailand	UNITE Database
T. sp. (SH1502450.08FU)	–	UDB013750	CP048	*Thelephora-Tomentella* EcM lineages	Thailand	UNITE Database
T. sp. (SH1853429.08FU)	–	UDB013771	CP077	*Thelephora-Tomentella* EcM lineages	Thailand	UNITE Database
T. sp. (SH1853842.08FU)	UDB023385	–	TU115846	*Thelephora-Tomentella* EcM lineages	Thailand	UNITE Database
T. sp. (SH1853922.08FU)	UDB023388	–	TU115858	*Thelephora-Tomentella* EcM lineages	Thailand	UNITE Database

T. sp. (SH1853923.08FU)	UDB023389	_	TU115879	*Thelephora-Tomentella* EcM lineages	Thailand	UNITE Database
T. sp. (SH1893848.08FU)	UDB023398	_	TU115878	*Thelephora-Tomentella* EcM lineages	Thailand	UNITE Database
T. sp. (SH1853934.08FU)	UDB023399	_	TU115897	*Thelephora-Tomentella* EcM lineages	Thailand	UNITE Database
T. sp. (SH1893915.08FU)	UDB023401	_	TU115903	*Thelephora-Tomentella* EcM lineages	Thailand	UNITE Database
T. sp. (SH1572167.08FU)	–	UDB014155	TU110886	*Thelephora-Tomentella* EcM lineages	Vietnam	UNITE Database
T. sp. (SH1572167.08FU)	UDB014160	_	TU110893	*Thelephora-Tomentella* EcM lineages	Vietnam	UNITE Database
T. sp. (SH1893908.08FU)	–	UDB014164	TU110897	*Thelephora-Tomentella* EcM lineages	Vietnam	UNITE Database
T. sp. (SH1888015.08FU)	–	UDB014165	TU110898	*Thelephora-Tomentella* EcM lineages	Vietnam	UNITE Database
T. sp. (SH1610992.08FU)	–	UDB014166	TU110899	*Thelephora-Tomentella* EcM lineages	Vietnam	UNITE Database
T. sp. (SH1610992.08FU)	UDB014167	_	TU110900	*Thelephora-Tomentella* EcM lineages	Vietnam	UNITE Database
T. sp. (SH1854223.08FU)	–	UDB014172	TU110905	*Thelephora-Tomentella* EcM lineages	Vietnam	UNITE Database
T. sp. (SH2040154.08FU)	–	UDB014173	TU110906	*Thelephora-Tomentella* EcM lineages	Vietnam	UNITE Database
T. sp. (SH1572167.08FU)	UDB014175	_	TU110908	*Thelephora-Tomentella* EcM lineages	Vietnam	UNITE Database

T. sp. (SH1642927.08FU)	–	UDB014177	TU110910	*Thelephora-Tomentella* EcM lineages	Vietnam	UNITE Database
T. sp. (SH1854004.08FU)	–	UDB014178	TU110911	*Thelephora-Tomentella* EcM lineages	Vietnam	UNITE Database
T. sp. (SH1642927.08FU)	UDB014179	_	TU110912	*Thelephora-Tomentella* EcM lineages	Vietnam	UNITE Database
T. sp. (SH2094508.08FU)	UDB014180	_	TU110914	*Thelephora-Tomentella* EcM lineages	Vietnam	UNITE Database
T. sp. (SH1894047.08FU)	–	UDB014182	TU110916	*Thelephora-Tomentella* EcM lineages	Vietnam	UNITE Database
T. sp. (SH1894031.08FU)	–	UDB014183	TU110917	*Thelephora-Tomentella* EcM lineages	Vietnam	UNITE Database
T. sp. (SH1893956.08FU)	–	UDB014216	TU110953	*Thelephora-Tomentella* EcM lineages	Vietnam	UNITE Database

REFERENCES

Disyatat, N.R., Yomyart, S., Sihanonth, P., and Piapukiew, J. (2016). Community structure and dynamics of ectomycorrhizal fungi in a dipterocarp forest fragment and plantation in Thailand. *Plant Ecol. Divers.* 9, 577–588. doi: 10.1080/17550874.2016.1264018

Ge, Z.W., Smith, M.E., Zhang, Q.Y., and Yang, Z.L. (2012). Two species of the Asian endemic genus *Keteleeria* form ectomycorrhizas with diverse fungal symbionts in southwestern China. *Mycorrhiza* 22, 403–408. doi: 10.1007/s00572-011-0411-1

Kaewgrajang, T., Sangwanit, U., Kodama, M., and Yamato, M. (2014). Ectomycorrhizal fungal communities of *Dipterocarpus alatus* seedlings introduced by soil inocula from a natural forest and a plantation. *J. Forest Res.* 19, 260–267. doi: 10.1007/s10310-013-0408-z

Kõljalg, U., Dahlberg, A., Taylor, A.F.S., Larsson, E., Hallenberg, N., Stenlid, J. et al. (2000). Diversity and abundance of resupinate thelephoroid fungi as ectomycorrhizal symbionts in Swedish boreal forests. *Mol. Ecol.* 9, 1985–1996. doi: 10.1046/j.1365-294X.2000.01105.x

Kuhar, F., Barroetaveña, C., and Rajchenberg, M. (2016). New species of *Tomentella* (Thelephorales) from the Patagonian Andes forests. *Mycologia* 108, 780–790. doi: 10.3852/15-244

Huang, J., Nara, K., Zong, K., Wang, J., Xue, S., Peng, K. et al. (2014). Ectomycorrhizal fungal communities associated with masson pine (*Pinus massoniana*) and white oak (*Quercus fabri*) in a manganese mining region in Hunan Province, China. *Fungal Ecol.* 9, 1–10. doi: 10.1016/j.funeco.2014.01.001

Ingleby, K., Thuy, L.T.T., Phong, N.T., and Mason, P.A. (2000).Ectomycorrhizal inoculum potential of soils from forest restoration sites in South Vietnam. *J. Trop. For. Sci.* 12, 418–422.

Larsson, K.H., Larsson, E., and Kõljalg, U. (2004). High phylogenetic diversity among corticioid homobasidiomycetes. *Mycol. Res.* 108, 983–1002. doi: 10.1017/S0953756204000851

Larsson, K.H., Svantesson, S., Miscevic, D., Kõljalg, U., and Larsson, E. (2019). Reassessment of the generic limits for *Hydnellum* and *Sarcodon* (Thelephorales, Basidiomycota). *MycoKeys* 54, 31–47. doi: 10.3897/mycokeys.54.35386

Lu, X., Mu, Y.H., and Yuan, H.S. (2018a). Two new species of *Tomentella* (Thelephorales, Basidiomycota) from Lesser Xingan Mts., northeastern China. *Phytotaxa* 369, 080–092. doi: 10.11646/phytotaxa.369.2.2

Lu, X., Steffen, K., and Yuan, H.S. (2018b). Morphological and molecular identification of three new species of *Tomentella* from Finland. *Mycologia* 110, 1–15. doi: 10.1080/00275514.2018.1474683

Morris, M.H., Smith, M.E., Rizzo, D.M., Rejmánek, M., and Bledsoe, C.S. (2008). Contrasting ectomycorrhizal fungal communities on the roots of co-occurring oaks (*Quercus* spp.) in a California woodland. *New Phytol.* 178, 167–176. doi: 10.1111/j.1469-8137.2007.02348.x

Nouhra, E., Pastor, N., Becerra, A., Areitio, E.S., and Geml, J. (2015). Greenhouse Seedlings of *Alnus* Showed Low Host Intrageneric Specificity and a Strong Preference for Some *Tomentella* Ectomycorrhizal Associates. *Microb. Ecol.* 69, 813–825. doi: 10.1007/s00248-014-0522-2

Peintner, U., and Dämmrich, F. (2012). *Tomentella alpina* and other tomentelloid taxa fruiting in a glacier valley. *Mycol. Prog.* 11, 109–119. doi: 10.1007/s11557-010-0734-x

Richard, F., Roy, M., Shahin, O., Sthultz, C., Duchemin, M., Joffre, R. et al. (2011) Ectomycorrhizal communities in a Mediterranean forest ecosystem dominated by *Quercus ilex*: seasonal dynamics and response to drought in the surface organic horizon. *Ann. Forest Sci.* 68, 57–68. doi: 10.1007/s13595-010-0007-5

Smith, M.E., Douhan, G.W., and Rizzo, D.M. (2007). Ectomycorrhizal community structure in a xeric *Quercus* woodland based on rDNA sequence analysis of sporocarps and pooled roots. *New Phytol.* 174, 847–863. doi: 10.1111/j.1469-8137.2007.02040.x

Suvi, T., Tedersoo, L., Abarenkov, K., Beaver, K., Gerlach, J., and Kõljalg, U. (2010). Mycorrhizal symbionts of *Pisonia grandis* and *P. sechellarum* in Seychelles: identification of mycorrhizal fungi and description of new *Tomentella* species. *Mycologia* 102, 522–533. doi: 10.3852/09-147

Tedersoo, L., Harend, H., Buegger, F., Pritsch, K., Saar, I., and Kõljalg, U. (2014). Stable isotope analysis, field observations and synthesis experiments suggest that *Odontia* is a non-mycorrhizal sister genus of *Tomentella* and *Thelephora*. *Fungal Ecol.* 11, 80–90. doi: 10.1016/j.funeco.2014.04.006

Tedersoo, L., Suvi, T., Larsson, E., and Kõljalg, U. (2006). Diversity and community structure of ectomycorrhizal fungi in a wooded meadow. *Mycol. Res.* 110, 734–748. doi: 10.1016/j.mycres.2006.04.007

Tedersoo, L., Suvi, T., Beaver, K., and Kõljalg, U. (2007). Ectomycorrhizal fungi of the Seychelles: diversity patterns and host shifts from the native *Vateriopsis seychellarum* (Dipterocarpaceae) and *Intsia bijuga* (Caesalpiniaceae) to the introduced *Eucalyptus robusta* (Myrtaceae), but not *Pinus caribea* (Pinaceae). *New Phytol.* 175, 321–333. doi: 10.1111/j.1469-8137.2007.02104.x

Wang, Q., Gao, C., and Guo, L.D. (2011). Ectomycorrhizae associated with *Castanopsis fargesii*(Fagaceae) in a subtropical forest, China. *Mycol. Prog.* 10, 323–332. doi: 10.1007/s11557-010-0705-2

Wang, Q., He, X.H., and Guo, L.D. (2012). Ectomycorrhizal fungus communities of *Quercus liaotungensis* Koidz of different ages in a northern China temperate forest. *Mycorrhiza* 22, 461–470. doi: 10.1007/s00572-011-0423-x

Yorou, N.S., and Agerer, R. (2008). *Tomentella africana*, a new species from Benin (West Africa) identified by morphological and molecular data. *Mycologia* 100, 68–80. doi: 10.1080/15572536.2008.11832499

Yorou NS., Diabaté M., and Agerer, R. (2012a). Phylogenetic placement and anatomical characterisation of two new West African *Tomentella* (Basidiomycota, Fungi) species. *Mycol. Prog.* 11, 171–180. doi: 10.1007/s11557-011-0739-0

Yorou, N.S., Gardt, S., Guissou, M.L., Diabaté, M., and Agerer, R. (2012b). Three new *Tomentella* species from West Africa identified by anatomical and molecular data. *Mycol. Prog.* 11, 449–462. doi: 10.1007/s11557-011-0760-3

Yorou, N.S., Guelly, A.K., and Agerer, R. (2011). Anatomical and ITS rDNA-based phylogenetic identification of two new West African resupinate thelephoroid species. *Mycoscience* 52, 363–375. doi: 10.1007/S10267-011-0117-4

Yorou N,S., Kõljalg, U., Sinsin, B., and Agerer, R. (2007). Studies in African thelephoroid fungi: 1. *Tomentella capitata* and *Tomentella brunneocystidia*, two new species from Benin (West Africa) with capitate cystidia. *Mycol. Prog.* 6, 7–18. doi: 10.1007/s11557-006-0519-4

Yuan, Y., Wu, F., Dai, YC., Qin, W.M., and Yuan, H.S. (2018). *Odontia aculeata* and *O. sparsa*, two new species of tomentelloid fungi (Thelephorales, Basidiomycota) from the secondary forests of northeast China. *Phytotaxa* 372, 183–192. doi: 10.11646/phytotaxa.372.3.1

Yuan, H.S., Lu, X., Dai, Y.C., Kevin, D.H., Kan, Y.H. et al (2020). Fungal diversity notes 1276–1386: taxonomic and phylogenetic contributions to fungal taxa. *Fungal Divers.* 1, 1–260. doi: 10.1007/s13225-020-00461-7
